# Tuning the Driving Force for Charge Transfer in Perovskite–Chromophore Systems

**DOI:** 10.1021/acs.jpcc.3c03815

**Published:** 2023-07-26

**Authors:** Zimu Wei, Jence T. Mulder, Rajeev K. Dubey, Wiel H. Evers, Wolter F. Jager, Arjan J. Houtepen, Ferdinand C. Grozema

**Affiliations:** Department of Chemical Engineering, Delft University of Technology, Van der Maasweg 9, 2629 HZ Delft, The Netherlands

## Abstract

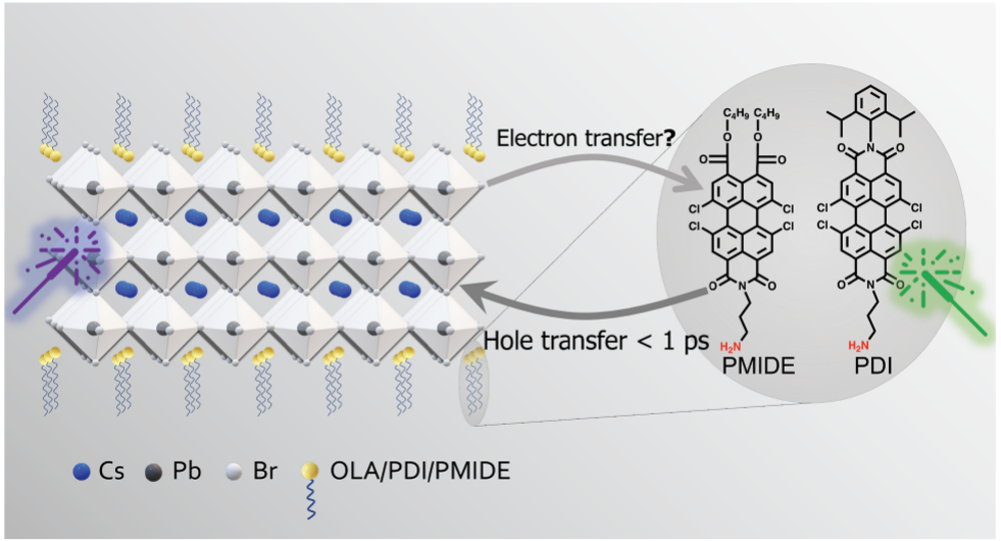

Understanding the interplay between the kinetics and energetics of photophysical processes in perovskite–chromophore hybrid systems is crucial for realizing their potential in optoelectronics, photocatalysis, and light-harvesting applications. By combining steady-state optical characterizations and transient absorption spectroscopy, we have investigated the mechanism of interfacial charge transfer (CT) between colloidal CsPbBr_3_ nanoplatelets (NPLs) and surface-anchored perylene derivatives and have explored the possibility of controlling the CT rate by tuning the driving force. The CT driving force was tuned systematically by attaching acceptors with different electron affinities and by varying the bandgap of NPLs via thickness-controlled quantum confinement. Our data show that the charge-separated state is formed by selectively exciting either the electron donors or acceptors in the same system. Upon exciting attached acceptors, hole transfer from perylene derivatives to CsPbBr_3_ NPLs takes place on a picosecond time scale, showing an energetic behavior in line with the Marcus normal regime. Interestingly, such energetic behavior is absent upon exciting the electron donor, suggesting that the dominant CT mechanism is energy transfer followed by ultrafast hole transfer. Our findings not only elucidate the photophysics of perovskite–molecule systems but also provide guidelines for tailoring such hybrid systems for specific applications.

## Introduction

In recent years, strenuous synthetic efforts toward colloidal perovskite nanomaterials have unlocked the potential of perovskite nanoplatelets (NPLs) for LEDs,^1−3^ lasers,^4,5^ luminescent solar concentrators,^6^ photovoltaics,^7^ and photocatalysis.^8,9^ They can be synthesized with thicknesses smaller than their exciton Bohr radius, showing strong quantum and dielectric confinement.^10,11^ Hence, they exhibit unique thickness-dependent absorption and emission with narrow line width and large exciton binding energy (i.e., 200–400 meV).^12,13^ The latter benefits the light-emitting properties of NPLs but limits their potential for photovoltaics and photodetectors by hampering the charge separation process.^14^ Our group has previously shown that efficient charge separation can be achieved by attaching strongly electron accepting chromophores onto the surface of colloidal CsPbBr_3_ NPLs.^15^ To further control and optimize charge separation in those hybrid systems toward specific applications, understanding the kinetics and energetics of charge transfer (CT) between the chromophoric molecules and perovskite nanomaterials is essential.

Despite some recent interest in perovskite–molecule hybrid systems,^16−18^ a photophysical understanding of such systems remains underdeveloped and sometimes disputed.^19,20^ In particular, systematic studies on how to control the CT rate by tuning the driving force are lacking. Most studies of perovskite–molecule systems focused on relatively large perovskite nanocrystals with bulklike electronic structures. In this weakly quantum confined regime, it becomes questionable to approximate the conduction band and valence band of NCs by two single levels when evaluating the interfacial charge transfer. This problem can be alleviated by the strong quantum confinement in NPLs.^21^ Through precise control over the number of monolayers, the bandgap energy of NPLs can be varied accordingly, thereby tuning the CT driving force. On the other hand, the CT driving force can be tuned by customizing the redox potential of molecules. By attaching different molecules to the NPLs synthesized in the same batch, we can study the effect of energetics while averting the reproducibility issue of nanomaterials.

In this work, we investigate the mechanism of interfacial CT between colloidal 2D CsPbBr_3_ NPLs (*n* = 3 and 4) and surface-anchored perylene derivatives, with a focus on the effect of driving force on the CT rate. Through the combination of steady-state optical characterizations and transient absorption measurements, we have established a detailed picture of the photophysical processes upon selectively exciting either the electron donor or acceptor in the same system. Upon exciting attached acceptor molecules, we demonstrate hole transfer from perylene derivatives to CsPbBr_3_ NPLs on a picosecond time scale. By only varying the electron affinity of the perylene derivatives, we show that the hole transfer rate increases with an increasing driving force. Strikingly, this energetic effect is absent when varying the NPLs bandgap, which is an indirect consequence of concomitant change in their lateral sizes. Upon exciting CsPbBr_3_ NPLs, our data suggest the driving force dependence of CT rate is incompatible with electron transfer in the Marcus normal regime. We conclude that the photoexcitation of NPLs predominately leads to energy transfer from NPLs to acceptor molecules, followed by ultrafast hole transfer to produce triplet states in the molecules. Our findings deepen the fundamental understanding of the photophysics of perovskite–molecule hybrid systems and pave the way for tailoring those hybrid systems for various light-harvesting applications.

## Methods

### Chemicals

Cesium carbonate (Cs_2_CO_3_, 99%, Sigma-Aldrich), lead(II) bromide (PbBr_2_, 99.999%, Sigma-Aldrich), oleic acid (OA, Extra Pure, Thermo Scientific Chemicals), oleylamine (OLA, approximate C18 content 80–90%, Thermo Scientific Chemicals), acetone (ACS reagent, ≥99.5%, Sigma-Aldrich), hexane (suitable for HPLC, ≥95%, Sigma-Aldrich), toluene, and dichloromethane (for spectroscopy Uvasol, Sigma-Aldrich) were used as received.

### Synthesis of CsPbBr_3_ NPLs

CsPbBr_3_ NPLs were synthesized following a procedure modified from a procedure described by Bohn et al.^22^ The Cs–oleate precursor solution was prepared by adding 0.2 mmol of Cs_2_CO_3_ powder in 20 mL of oleic acid. The PbBr_2_ precursor solution was prepared by adding 0.5 mmol of PbBr_2_ powder with 500 μL of oleylamine and 500 μL of oleic acid in 50 mL of toluene. Both precursor solutions were stirred continuously at 100 °C until all the powders were dissolved. 150 μL of the Cs–oleate precursor solution was added into PbBr_2_ precursor solution (1.5 mL for 4ML and 3 mL for 3ML) under vigorous stirring. After 5 s, 2 mL of acetone was quickly added. After 1 min, the solution was centrifuged at 4000 rpm for 3 min. The precipitate was redispersed in hexane. All CsPbBr_3_ NPLs were synthesized at room temperature and under ambient conditions.

### Preparation of Sample Solutions (NPLs + PDI/PMIDE)

The syntheses of PDI and PDI-D have been published in refs (15) and (23), and the synthesis of PMIDE is provided in the Supporting Information. The diluted NPLs solution in hexane was added into three cuvettes, each containing 2.7 mL. Subsequently, 150 μL of 10^–4^ M PDI or PMIDE in DCM was added to form NPLs–chromophore hybrids at room temperature. For the reference NPLs solution, 150 μL of DCM was added into the cuvette. The same sample solution was used for absorption, PL, PL lifetime, and TA measurement on the same day. The mixed solution was filtered and sonicated for 2 min before the TA measurement.

### Cyclic Voltammetry

Electrochemical behavior of the compounds was studied using cyclic voltammetry (Autolab PGSTAT128N potentiostat) in a three-electrode single-compartment cell consisting of a platinum sheet, evaporated on quartz, as the working electrode, a silver wire as the pseudoreference electrode, and a platinum sheet as the counter electrode. The cell was connected to the computer-controlled potentiostat (Autolab PGSTAT128N potentiostat). Anhydrous DCM containing 0.1 M tetrabutylammonium hexafluorophosphate was used as an electrolyte solution. The measurements were inside a nitrogen filled glovebox. The concentrations of the prepared samples were sufficiently low. Under these experimental conditions, the ferrocene oxidation was observed at 0.455 (PDI)/0.43 (PMIDE) V. The potentials of all the reversible peaks are reported as *E*_1/2_ = (*E*_p_^a^ + *E*_p_^c^)/2 in V vs Fc/Fc^+^ and quoted to the nearest 0.01 V. The measurements were performed at a 0.01 V s^–1^ scan rate. The energy level of LUMO was calculated as *E*_LUMO_ = −(*E*_1red_ + 4.8 eV). The energy level of HOMO was calculated as *E*_HOMO_ = *E*_*L*UMO_ – *E*_g_. The optical band gap, *E*_g_, was estimated from absorption onset wavelength *E*_g_ = 1240/λ.

### Transmission Electron Microscopy (TEM)

TEM images were acquired using a JEOL JEM-1400 plus TEM microscope operating at 120 kV. TEM samples were prepared by drop-casting NPLs hexane solution onto a carbon-coated Cu TEM grid. High-resolution TEM (HR-TEM) images were acquired using a JEOL JEM-3200 FSC operating at 300 kV and a Gatan K2 camera operating in counting mode. The images were taken at zero loss with a slit with 20 eV.

### Optical Spectroscopy

Absorption spectra were measured by a PerkinElmer Lambda 365 UV–vis spectrophotometer. PL spectra were measured by Edinburgh Instruments FLS980 spectrofluorometer with a xenon lamp as excitation source. PL lifetimes were recorded on an Edinburgh LifeSpec-ps spectrometer with a fixed excitation wavelength of 404 nm.

### Femtosecond Transient Absorption Measurement

For fs-TA measurements, the pump beam was generated by a YB-KGW oscillator (Light Conversion, Pharos SP-06-200) operating at 5 kHz (chopped at 2.5 kHz) with a pulse duration of 180 fs. The pump wavelength was tuned by sending the fundamental beam (1028 nm) through an optical parametric amplifier (Light Conversion, ORPHEUS-PO15F5HNP1). The probe beam was generated by focusing a small fraction of the fundamental beam in a CaF_2_ crystal, producing a broadband continuum spectrum (350–910 nm). The time delay (up to 3 ns) between the pump and probe was controlled by a physical delay stage. The difference in the absorption (Δ*A* = log(*I*_pump-on_/*I*_pump-off_)) was acquired by using a commercial TA spectrometer (HELIOS, Ultrafast Systems). The sample solution was measured in a 2 mm quartz cuvette under vigorous stirring at the magic angle (54.7°). The pump power stayed stable during each measurement with a deviation smaller than 2% before and after each measurement. To ensure the sample stability, at least 3 scans were performed for each measurement; no degradation was detected during the TA measurement.

## Results and Discussion

### System Design

**Figure 1:**
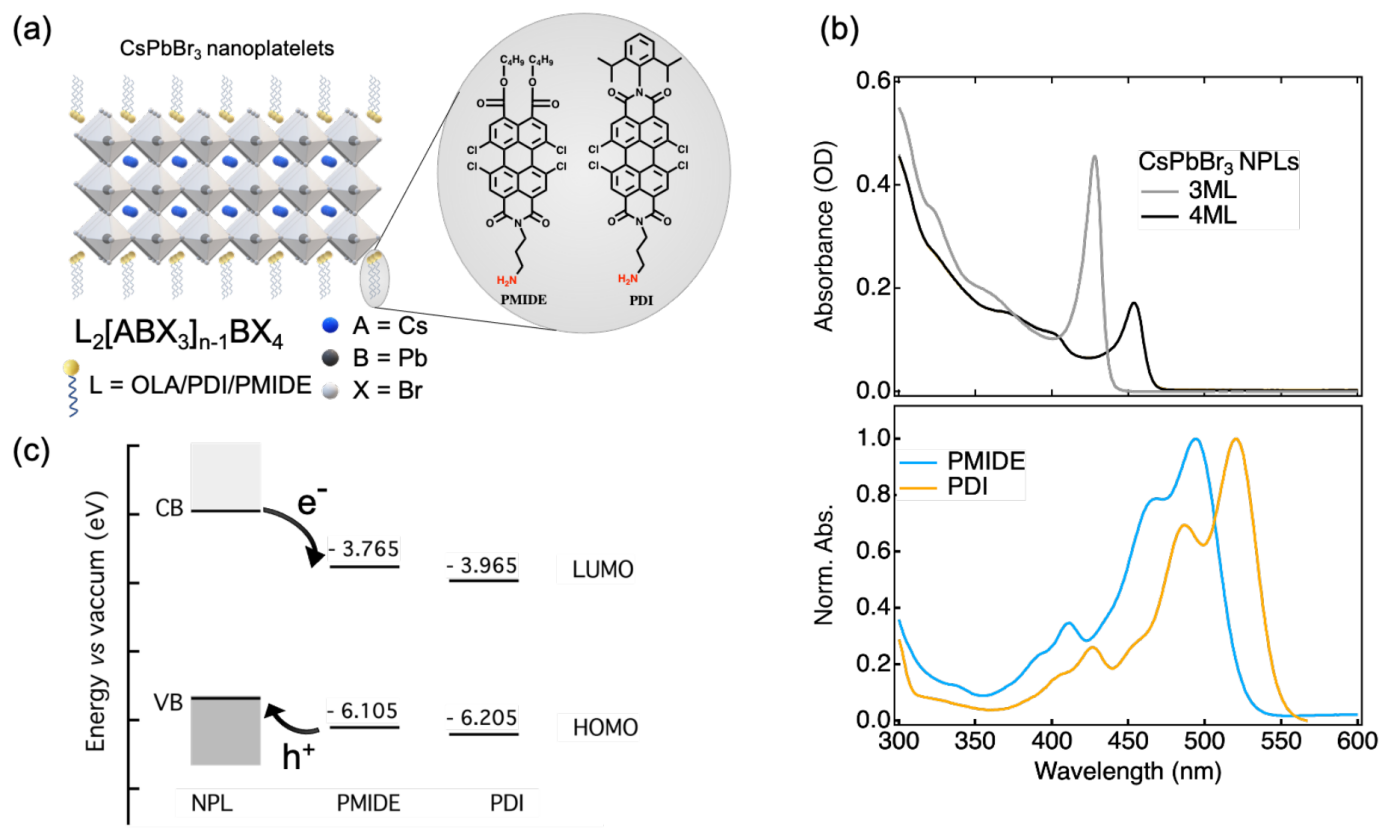
System design of perovskite–chromophore hybrids. (a) Schematic representation of CsPbBr_3_ nanoplatelets (NPLs)–perylene derivative hybrids. (b) Absorption spectra of CsPbBr_3_ NPLs with different thicknesses in hexane and normalized absorption spectra of perylene monoimide (PMIDE) and of perylene diimide (PDI) in dichloromethane (DCM). (c) Estimated energy levels of the CsPbBr_3_ NPLs and molecular acceptors. Arrows indicate interfacial electron transfer and hole transfer pathways.

To design a proper donor–acceptor system, suitable energetic and spectroscopic properties are essential. Therefore, we chose a system consisting of CsPbBr_3_ NPLs functionalized with with either perylene diimide derivatives (PDI) or perylene monoimide diester derivatives (PMIDE) (Figure 1a). Details of syntheses are provided in the Supporting Information. Both chromophores are excellent electron acceptors but with different electron accepting abilities.^24^ As both molecules are terminated with the same alkylamine group at the imide position, this allows us to study the effect of driving force on CT without changing the anchoring group or the CT distance. On the other hand, the CT driving force can be tuned by varying the bandgap of CsPbBr_3_ NPLs via thickness-controlled quantum confinement.^11,25^ By controlling the synthesis conditions, colloidal CsPbBr_3_ NPLs with monodispersed thickness are obtained.^22^ Their thickness-dependent absorption properties are listed in Figure 1b. Based on the TEM images (Figure S1), their sharp absorption peaks at 428 nm (2.90 eV) and 454 nm (2.73 eV) are assigned to the excitonic absorption of 3 monolayer (∼1.7 nm) and 4 monolayer (∼2.5 nm) NPLs,^26^ respectively. 3ML and 4ML NPLs were chosen because of their relatively good stability and minimized spectral overlap with the absorption of PDI and PMIDE (Figure 1b). In this way, we can selectively excite both components at different wavelengths to explore the effect of driving force on both electron transfer from NPLs to PDI/PMIDE and hole transfer from PDI/PMIDE to NPLs.

The estimated energy diagram of the perovskite–chromophore system is shown in Figure 1c. The energy levels of HOMO and LUMO of PMIDE and PDI are determined based on the electrochemical characterization (Figure S2). Experimental details regarding the cyclic voltammetry are described in the Supporting Information. Unfortunately, it is impractical to use the same method to determine the band edge positions for CsPbBr_3_ NPLs due to their poor electrochemical stability. Although there have been some attempts to estimate the energy levels of conduction and valence band of CsPbBr_3_ NPLs,^16^ a large uncertainty, on the order of hundreds of meV, is typically expected. However, it is still possible to make a few qualitative estimations that can reflect the energetic environment of our hybrid system. First, based on the reported VB edge positions (−5.4 eV) of ∼10 nm CsPbBr_3_ nanocrystals,^27^ strongly quantum-confined CsPbBr_3_ NPLs are expected to have a deeper VB level. This gives rise to an upper limit of 0.8 eV for the driving force of hole transfer. Second, because the hole transfer from PDI/PMIDE to CsPbBr_3_ NPLs is energetically favored as evident in our previous report^15^ and results in this work, the lower limit of the VB energy level is −6.1 eV. Finally, the energy difference between the CB and VB should be larger than the energy of the exciton peak. As a result, the driving force for electron transfer is at least 0.4 and 0.6 eV for 4ML NPLs and 3ML NPLs, respectively. Therefore, the hybrid system should possess a type II energy level alignment, as shown in Figure 1c.

### Steady-State Optical Properties

**Figure 2:**
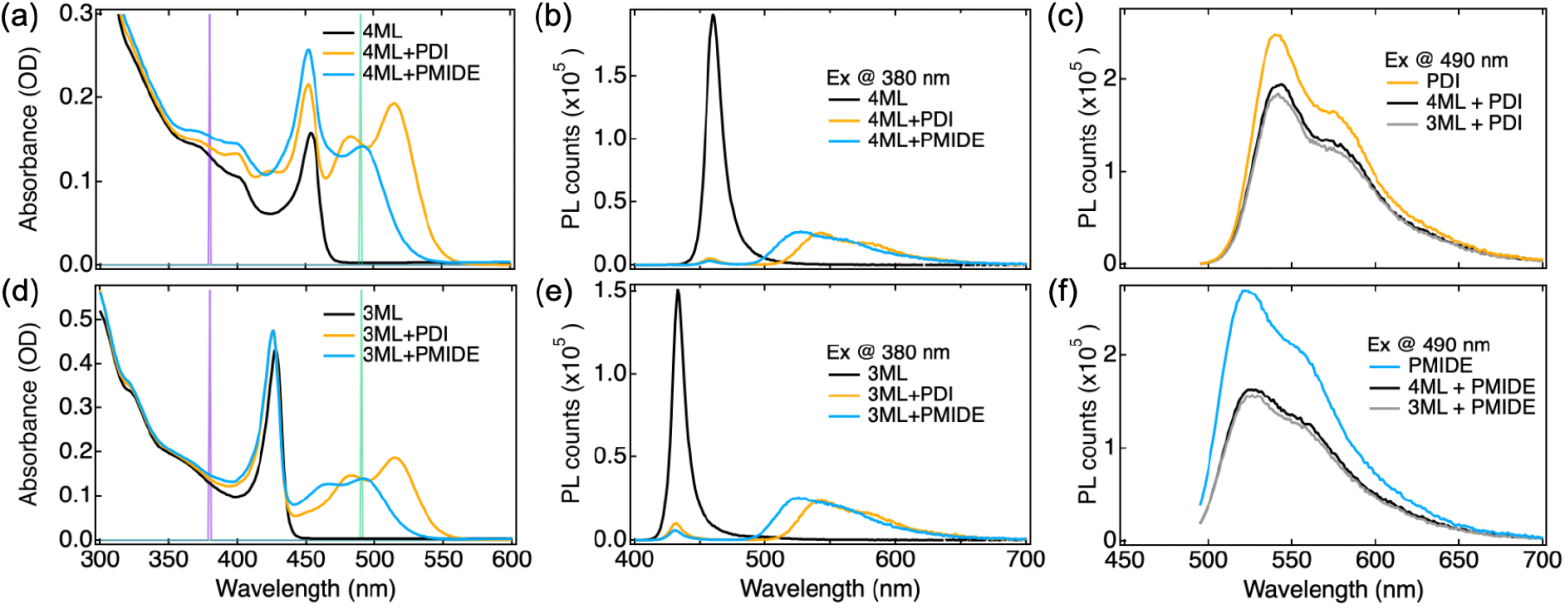
Optical absorption and photoluminescence emission spectra in a Hex:DCM (18:1) mixture with *c*_PDI/PMIDE_ ≈ 5 μM. (a, d) Absorption spectra of CsPbBr_3_ NPLs, NPLs + PDI, and NPLs + PMIDE (4ML in (a) and 3ML in (d)). (b, d) Emission spectra of NPLs, NPLs + PDI, and NPLs + PMIDE excited at 380 nm. (e) Emission spectra of PDI and NPLs + PDI excited at 490 nm. (f) Emission spectra of PMIDE and NPLs + PMIDE excited at 490 nm. Purple and green lines indicate the excitation wavelengths (380 and 490 nm).

Hybrid systems of NPLs and acceptor molecules were produced by adding the PDI or PMIDE stock solutions in DCM to the CsPbBr_3_ NPLs solution in hexane. With this ligand exchange process, the PDI or PMIDE molecules were attached to the NPLs surfaces, presumably via the alkylammonium group. The direct proof of the attachment via the alkylammonium group is shown by the TA measurements, as described below. Considering the possible instability of CsPbBr_3_ NPLs in the polar solvent, the same amount of DCM was added to the CsPbBr_3_ NPLs solution in hexane to be used as the reference NPLs solution. Figure S3 clearly shows that the addition of DCM has a negligible impact on the optical properties of NPLs except for a minor reduction in the PL quantum yield, which is likely due to dissolution of some surface ligands. As shown in Figures 2a and 2d, upon addition of the acceptor molecules, the absorption spectrum of the hybrid system is basically a linear combination of the individual spectra of the two components. This suggests a minimal electronic coupling between donor and acceptor in the ground state. Thus, no CT-like complex is formed in the ground state. Based on the extinction coefficients of PDI and PMIDE (Figure S4), the concentrations of both are estimated to be ∼5 μM in the mixed solution.

Figures 2b and 2e show the PL spectra of the NPLs and the hybrid systems. Notably, the PL of both 3ML and 4ML NPLs is almost entirely quenched in the presence of acceptor molecules. In addition to the reduced PL quantum yield, their PL decays significantly faster upon the addition of acceptor molecules (Figure S5). This is a strong indication of either electron transfer or energy transfer from the NPLs to PDI or PMIDE. To evaluate the possibility of energy transfer, PL excitation spectra were measured for the 4ML + PDI and 4ML + PMIDE hybrid systems. By monitoring the emission intensity at 550 nm while scanning the excitation wavelength from 350 to 545 nm, we can clarify the origin of the PDI/PMIDE emission in the hybrid systems. If this emission originates from the energy transfer from 4ML NPLs, the spectral feature of NPLs absorption is expected to be present in the PL excitation spectrum. However, no such response was observed for either of the hybrid systems, as the excitation spectra of hybrid systems nicely overlap with the absorption spectra of reference PDI/PMIDE molecules in solution (Figure S6a). Considering a less likely energy transfer from 3ML NPLs due to a smaller overlap between the PL of 3ML NPLs and the absorption of PDI/PMIDE (Figure S6b), the PDI/PMIDE emission in Figures 2b and 2e should only stem from the photoexcited PDI/PMIDE instead of the energy transfer from NPLs to PDI/PMIDE. Furthermore, upon excitation at 490 nm (Figures 2c and 2f), PDI/PMIDE emission in the hybrid systems is also quenched, indicating hole transfer from the PDI/PMIDE to NPLs. Note that although no evidence of energy transfer from NPLs to PDI/PMIDE was found based on their steady-state optical properties, it is impossible to exclude the possibility of energy transfer followed by hole transfer from PDI/PMIDE to NPLs on an ultrafast time scale.

### Photoexcitation at 490 nm

To investigate whether there is hole transfer from PDI/PMIDE to the NPLs and how it may be affected by the driving force, the excited-state dynamics of the hybrid systems were measured by femtosecond transient absorption (TA) spectroscopy, exciting at 490 nm (see the Supporting Information for details). Because the photon energy of the pump pulses was below the bandgap of the NPLs, only the acceptor molecules were excited. To rule out the possibility of sub-bandgap absorption, we measured the TA spectra of a reference 4ML NPLs solution excited at 490 nm under different pump fluences, covering the pump fluence used for the main text. Indeed, apart from the common coherent artifact during the pump–probe overlap (<0.5 ps), no TA signal was detected for the reference NPLs (Figure S7).

**Figure 3:**
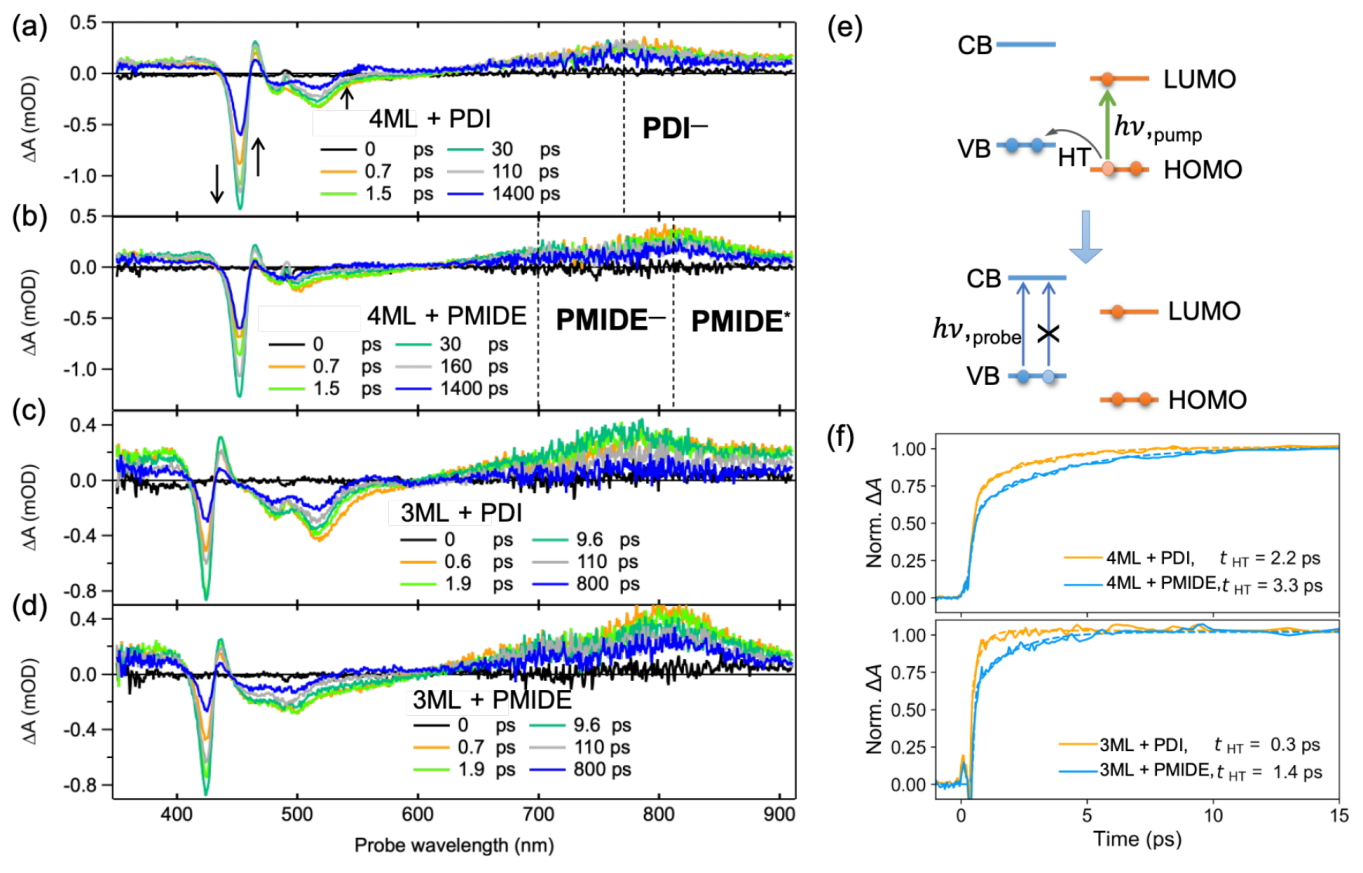
Transient absorption spectra of perovskite–chromophore hybrid systems upon photoexcitation at 490 nm ((1.1–1.6) × 10^13^ photons/cm^2^/pulse). (a–d) TA spectra of 4ML NPLs + PDI, 4ML NPLs + PMIDE, 3ML NPLs + PDI, and 3ML NPLs + PMIDE. (e) Schematic of hole bleach induced by hole transfer upon photoexciting acceptor molecules. (f) Normalized kinetics at the wavelength of exciton bleach for NPLs and their fits in the time window of the hole transfer process.

**Figure 4:**
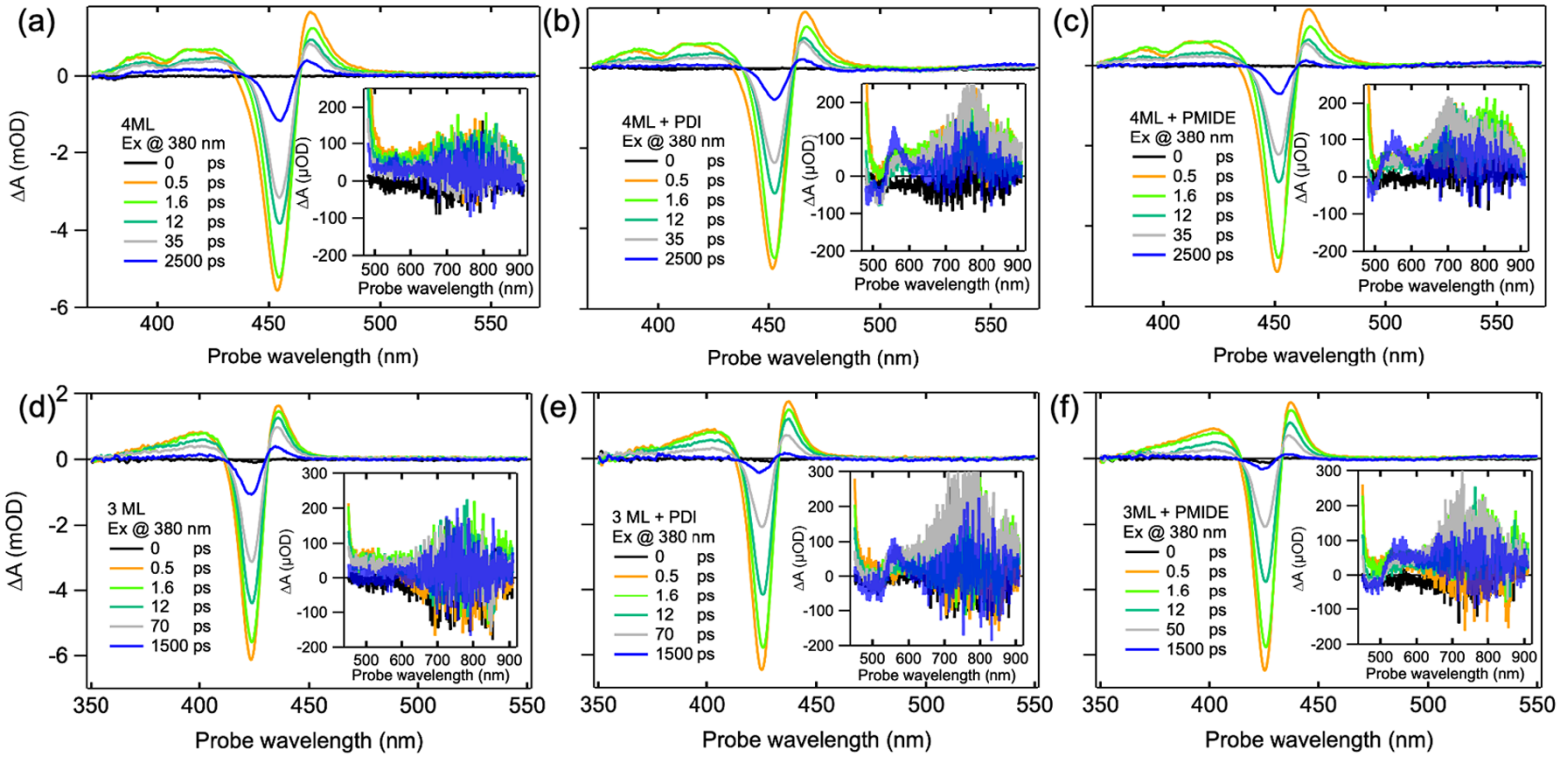
Transient absorption spectra upon photoexcitation at 380 nm (∼5 × 10^12^ photons/cm^2^). (a) TA spectra of 4ML NPLs. (b) TA spectra of 4ML NPLs + PDI. (c) TA spectra of 4ML NPLs + PMIDE. (d) TA spectra of 3ML NPLs. (e) TA spectra of 3ML NPLs + PDI. (f) TA spectra of 3ML NPLs + PMIDE. Insets are enlarged plots of TA spectra in the near-IR region.

The TA spectra of the hybrid systems upon photoexcitation at 490 nm are shown in Figure 3a–d. For the 4ML NPLs + PDI system (Figure 3a), immediately after the photoexcitation (at 0.7 ps), we observe the ground-state bleach (GSB) of PDI molecules at ∼520 nm. The broad induced absorption centered at ∼760 nm signals the presence of the PDI anion (PDI^–^), which is known to be blue-shifted with respective to the absorption of its excited state (PDI*) at ∼800 nm (Figure S8a).^28^ The TA spectrum between 350 and 470 nm exhibits typical features for CsPbBr_3_ NPLs (also see in Figure 4a), namely a sharp exciton bleach at ∼450 nm and two nearby induced absorption features.^13,16^ These spectral features are clear evidence of the hole transfer from the HOMO of PDI to the VB of NPLs, forming PDI anions and the hole bleach in the NPLs as illustrated in Figure 3e. With increasing time, we see the further growth of the NPLs exciton bleach and its nanosecond lifetime (Figure S9), indicating the formation of long-lived charges in the NPLs. Such hole transfer clearly also takes place in the 4ML NPLs + PMIDE system (Figure 3b), despite the fact that the PMIDE is a weaker electron acceptor. In this case, the two induced absorption bands are visible in the TA spectra. As compared with the TA spectra of the reference PMIDE (Figure S8b), the absorption band at ∼810 nm can be ascribed to the absorption of the PMIDE excited state (PMIDE*). Accordingly, we assign the absorption band at ∼700 nm to be the PMIDE anion (PMIDE^–^) absorption.^29^ The red-shifted PMIDE^–^ absorption as compared to the literature likely arises from the twisting of the perylene core due to the introduction of chlorides in the bay area. Similar to the 4ML NPLs, the hole transfer processes from both acceptor molecules to the 3ML NPLs are also neatly captured in their TA spectra (Figures 3c and 3d), including the formation of hole bleach of 3ML NPLs at ∼425 nm and the absorption of PDI and PMIDE anions. To verify whether the alkylammonium group functions as the anchoring group in attaching molecules to the NPLs surface, we performed TA measurements with a reference PDI molecule in which the propylamine group at the imide position is substituted by a diisopropylphenyl group. As evident in Figure S10, hole transfer from PDI to NPLs diminishes in the absence of a proper anchoring group. Based on this, we conclude that the acceptor molecules are attached to the NPLs surface via the alkylammonium group.

Because hole transfer takes place in all four hybrid systems, the next step is to quantify the hole transfer rate and to understand how it is influenced by the driving force. Considering the partial overlap between the absorption of PDI^–^/PMIDE^–^ and PDI*/PMIDE* and the concurrent absorption from unattached PDI/PMIDE molecules in solution, the formation of the NPLs hole bleach is a reliable indicator to reflect the hole transfer rate. Figure 3f shows the normalized single-wavelength fitting of the formation of NPLs bleach due to hole transfer. Details of the fitting procedure can be found in the Supporting Information. Overall, the hole transfer rates in all hybrid systems take place on the order of a picosecond. For both 4ML and 3ML systems, the hole transfer from the stronger electron acceptor, PDI, is faster than that from the weaker electron acceptor, PMIDE. In this comparison, both molecules are attached to the same NPLs via the same anchoring group. Therefore, other factors, such as the electronic coupling and the CT distance, should not play any role in determining the hole transfer rate. In addition, the effect of energetic distribution of band states in NPLs is insignificant due to the strong quantum confinement. This is supported by the calculations on band structures of CsPbBr_3_ NPLs, where the energy difference between the first and second confined energy levels is considerably large already for the 4ML.^21^ Furthermore, the fact that the spectra of formed NPLs bleach do not change in shape over the entire time (Figure S11) is consistent with the formation of holes in the NPLs directly at the band edge of the VB without subsequent relaxation. Hence, the increasing hole transfer rate with the increasing driving force conforms to CT in the Marcus normal regime.^30^

Interestingly, such Marcus normal behavior does not pertain to the comparison between 4ML and 3ML systems for the same acceptor molecules. By reduction of the thickness of NPLs, the VB of 3ML NPLs is expected to be lower than that of the 4ML NPLs due to quantum confinement, thereby lowering the CT driving force. To estimate the difference in the bandgap energy between 3ML and 4ML, we obtained the exciton energy and the exciton binding energy from their optical absorption spectra (Figure S12). Using this bandgap, we estimate the difference in the HT driving force between 3ML and 4ML to be approximately 95 meV (see the Supporting Information for detailed estimations). Nevertheless, hole transfer from acceptor molecules to the 3ML NPLs is much faster than that to the 4ML NPLs as demonstrated in Figure 3f. Given that hole transfer is expected to happen in the Marcus normal regime as described above, other factors unrelated to energetics must play a role. We have noticed that the average lateral sizes of the 3ML NPLs are almost 30 times larger than those of the 4ML NPLs, as indicated by their TEM images (Figure S13). Such a large difference in lateral sizes can have several consequences on the CT rate. First, the electronic coupling (*H*_DA_) in the Marcus theory is known to be surface-area-dependent in semiconductor nanoparticle–molecule systems.^31^ However, a smaller CT rate for a larger surface area is generally reported due to more delocalized wave function for larger sizes.^31,32^ Hence, the surface-area dependence of the electronic coupling is unlikely to be the reason for faster hole transfer to 3ML NPLs. The second is the reorganization energy. Although the reorganization energy of NPLs (λ_NPLs_) should be size-dependent, we expect the total reorganization energy of the hybrid systems (λ) to be dominated by the reorganization of the acceptor molecules (λ_PDI/PMI_), given the much larger sizes and more delocalized orbitals in NPLs. Hence, the total reorganization energy is expected to be similar for hybrid systems with the same chromophore. Finally, unlike conventional donor–acceptor systems, the CT rate in a nanoparticle–molecule system is expected to be proportional to the average number of molecules attached on each NPL.^16,18^ As a result of much larger lateral sizes, the average number of molecules attached on 3ML NPLs is estimated to be 15 times larger than that of 4ML NPLs (detailed estimation in the Supporting Information). Unfortunately, controlling the lateral dimensions of perovskite NPLs still remains challenging in the field.^10^ By tentatively plotting how hole transfer rate may scale with a prefactor in the context of Marcus theory (Figure S14), we reason that the abnormal driving-force dependence of hole transfer from the same molecules to 3ML/4ML NPLs is dominated by the different number of molecules attached as a consequence of very different lateral sizes.

### Photoexcitation at 380 nm

To determine whether PL quenching of NPLs is indeed due to electron transfer from NPLs to acceptor molecules and its driving force dependence, the TA spectra of the NPLs were measured upon photoexcitation at 380 nm. At this wavelength, the direct excitation of acceptor molecules is negligible given the similar absorbance of hybrid systems and reference NPLs (Figures 2a and 2d). All TA experiments were performed under sufficiently low pump fluences (∼5 × 10^12^ photons/cm^2^) to avoid multiexciton generation and photodegradation as much as possible. As shown in Figures 4a and 4d, the TA spectra of reference NPLs exhibit characteristics of photoexcited CsPbBr_3_ NPLs as reported in the literature,^13,16^ including the main exciton bleach due to the state filling of band edge excitons (∼450 nm for 4ML, ∼425 nm for 3ML) and two absorption features on the blue and red sides of the exciton bleach. Upon attachment of acceptor molecules, three drastic changes are seen in their TA spectra (Figures 4b,c and 4e,f). The obvious one is the faster recovery of NPLs bleach in hybrid systems. This is in good agreement with the quenched PL quantum yield and the shortened PL lifetime of NPLs as shown in Figures 2 and S5. The second change is observed in the near-IR region, where the GSB of PDI/PMIDE and their anion absorption (PDI^–^/PMIDE^–^) appear after tens of picoseconds, indicating the formation of the charge-separated (CS) state. Finally, on the nanosecond time scale, the absorption of PDI^–^/PMIDE^–^ decays accompanied by the growth of an induced absorption peak at ∼550 nm, while the GSB of PDI/PMIDE almost remains the same. Because the T_1_ → T_n_ transition of the PDI triplet has been reported to have a characteristic absorption at 555 nm,^33^ we attribute this induced absorption to the triplet state of the acceptor molecules. Given the high reactivity of the triplet state with oxygen molecules to generate singlet oxygen, a reactive species for photocatalysis, this intriguing finding demonstrates the potential of such hybrid systems for catalyst applications.^34^ Based on these observations, the sequence of photophysical processes upon excitation at 380 nm can be summarized as follows: (1) the photogeneration of excitons in NPLs, (2) the formation of a CS state, and (3) the triplet formation during charge recombination. Therefore, the faster decay of the excitons in NPLs is indeed associated with the charge transfer process. Nonetheless, it is still difficult to determine whether the CS state is formed due to direct electron transfer from the CB of NPLs to the LUMO of PDI/PMIDE or due to energy transfer from NPLs to PDI/PMIDE, followed by the ultrafast hole transfer process, because both pathways are energetically favorable and give rise to the same final products.

To further understand the photophysics upon excitation at 380 nm and to quantify the rate constants of the relevant photophysical processes, the TA spectra were fitted with global and target analysis using the open source software Glotaran.^35^ This method was used because the single-wavelength fitting of PDI/PMIDE anion absorption was inadequate to obtain reliable CT rates due to the noise in the near-infrared part of the spectra. With global and target analysis, the full 2D TA data were fitted to a predefined kinetic model, resulting in a series of spectra corresponding to species used in the kinetic model and their lifetimes (details are described in the Supporting Information). The global analysis of reference 4ML (3ML) NPLs yields three decay components with time constants of 0.62 (0.64) ps, 14 (29) ps, and 2.48 (1.62) ns (Table S1). The first subpicosecond time constant is in line with the reported cooling rates of hot carriers/excitons in 2D perovskite nanoplatelets (0.2–0.9 ps).^36,37^ Because photoexcitation at 380 nm is well above the bandgap of the NPLs, we assign the first decay component to the fast cooling of the hot excitons. This is followed by a decay component on the order of tens of picoseconds, which should correspond to fast trapping due to surface defects on perovskite NPLs.^38^ Lastly, the nanosecond component is comparable to the PL lifetime of the NPLs excitons (Figure S5).

**Figure 5:**
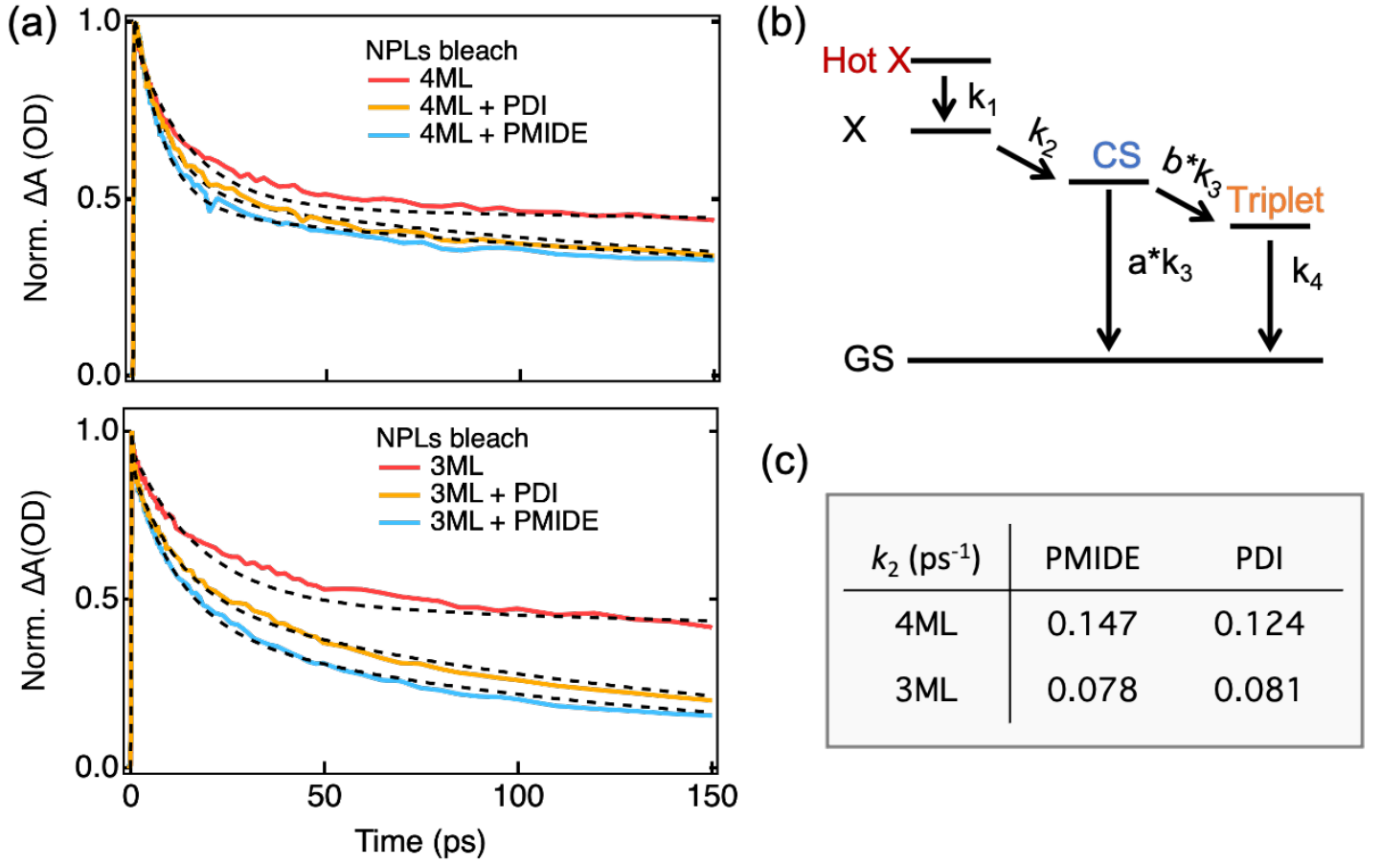
Global and target analysis of transient absorption spectra. (a) Normalized kinetics of exciton bleach of NPLs and their fits. (b) Kinetic model of target analysis for perovskite–chromophore systems. (c) Charge separation rates of all perovskite–chromophore systems excited at 380 nm.

Upon the addition of acceptor molecules, the faster decay of NPLs bleach is clearly evident in the comparison of their normalized kinetics, as shown in Figure 5a. Notably, the bleach of the NPLs recovers faster with the weaker acceptor PMIDE than with the stronger acceptor PDI for both 4ML and 3ML NPLs. This can be an indication of the occurrence of energy transfer from NPLs to PDI/PMIDE, as the spectral overlap is larger for the PMIDE molecules than for the PDI molecules (Figure S6b). On the other hand, it can also be a result of electron transfer from NPLs to PDI/PMIDE taking place in the Marcus-inverted region, where the electron transfer rate decreases with the increasing driving force.^30^ However, this is unlikely to be the responsible mechanism due to Auger-assisted mechanism that can effectively bypass the inverted region of electron transfer by dissipation of energy through intraband excitation, rather than by coupling to the vibrational density of states.^39−41^ In fact, despite extensive studies of charge transfer from colloidal semiconductor nanomaterials to adsorbed molecules using time-resolved spectroscopy, the inverted region was never observed for the charge transfer involving Coulomb-coupled charges (i.e., excitons).^42^ This is precisely the case for the electron transfer from NPLs to acceptor molecules studied in this work. To further validate this interpretation, the TA data of hybrid systems were fitted by the target analysis using the kinetic model illustrated in Figures 5b and S15. According to this model, the hot exciton in NPLs is generated upon photoexcitation, followed by rapid cooling to the band edge. Subsequently, the relaxed exciton undergoes charge transfer, forming a charge-separated (CS) state. Finally, the CS state can either decay back to the ground state or form the triplet state. The obtained rate constants of the different processes are summarized in Table S2. The validity of this analysis can be assessed by a comparison between the temporal kinetics and their fits at selected wavelengths and the resulting species-associated spectra. The former is an indicator of the fitting quality, while the interpretability of the latter should agree with the kinetic model used. Overall, the fitting quality is reasonable at various wavelengths in all the samples (Figures S16–S19). In the species-associated spectra, the evolution of the second species (relaxed exciton) to the third species (CS state) shows the emergence of PDI/PMIDE anion absorption at 760/700 nm, in line with a CT process from NPLs to PDI/PMIDE acceptors. Hence, the second rate constant (*k*_2_) should correctly reflect the CT rate. Although *k*_2_ is possibly influenced by the fast trapping in the NPLs, such an effect should be similar, if not identical, when comparing CT from the same NPLs to different molecules. As displayed in Figure 5c, the target analysis suggests the CT rate from 4ML NPLs to PMIDE is indeed faster than that to PDI, consistent with the faster decay of NPLs bleach with PMIDE. Nonetheless, in spite of a larger difference in the decay of NPLs bleach, such a trend becomes obscure for CT from 3ML NPLs, as both CT rates to PDI and to PMIDE are virtually the same. This seems to imply a slower energy transfer from 3ML NPLs to PDI compensated by the following faster hole transfer from PDI to 3ML NPLs. Although we acknowledge that the target analysis fits to the hybrid systems with 3ML NPLs are not as satisfactory as the 4ML due to the noisiness in the near-IR region, it is still possible to discern a generally faster rate for CT from 4ML NPLs than that from 3ML NPLs as indicated by the faster GSB formation of PDI/PMIDE for 4ML NPLs (Figure S20). This is interesting because an opposite trend has been demonstrated for the hole transfer, for which the smaller CT driving force for 3ML NPLs is overcompensated by a much larger number of molecules attached as compared to the 4ML NPLs. Because the electron transfer in the Marcus-inverted region should also be proportional to the number of acceptors attached, a faster CT rate is expected for the 3ML NPLs. However, in the case of energy transfer followed by hole transfer, the PL of 3ML NPLs indeed has a lower quantum yield (Figures 2b and 2e) as well as a smaller spectral overlap with the absorption of acceptor molecules (Figure S6b), thereby limiting the overall CT rate. In addition, the small amplitude of anion absorption in Figure 4 is in agreement with expectedly low PLQY of NPLs (<40%). Therefore, it is plausible that the energy transfer followed by hole transfer is the dominant pathway for forming the CS state upon excitation at 380 nm.

## Conclusion

In conclusion, we have systematically explored the mechanism of charge transfer between CsPbBr_3_ perovskite NPLs and perylene derivatives, especially to understand how we can control the charge transfer rate by tuning the driving force. The CT driving force has been tuned by attaching acceptor molecules with different electron affinities and by varying the bandgap of NPLs via thickness-controlled quantum confinement. The transient absorption data unambiguously show the necessity of an anchoring group for achieving charge separation. For perylene derivatives ending with an alkylammonium group, CS states are formed by selectively exciting either the electron donors or acceptors in the same system. This offers direct insight into the band edge positions of perovskite NPLs that cannot be determined electrochemically, owing to their notorious instability. Upon exciting acceptors at 490 nm, the stronger acceptor indeed leads to faster hole transfer from the acceptors to the perovskite NPLs. For NPLs with different bandgaps, the effect of the driving force on hole transfer is overshadowed by the difference in the number of attached molecules due to different lateral sizes of NPLs. Upon excitation of NPLs at 380 nm, we found the CT mechanism is distinct from a direct electron transfer from NPLs to acceptors in the Marcus normal regime. By carefully examining the TA data for all hybrid systems, we conclude that the dominant mechanism for CT is energy transfer from NPLs to acceptors, followed by an ultrafast hole transfer from acceptors to NPLs. These results highlight how the nature of CT between inorganic NPLs and molecular acceptors can be unraveled by a systematic variation. In addition to elucidating the photophysical picture in perovskite–chromophore systems, we have demonstrated the potential of such hybrid systems for triplet sensitization. With these results, we envision that functionalizing the surface of 2D perovskite nanomaterials is not only a successful strategy for deepening the fundamental understanding of intermolecular CT in such systems. This increased understanding is of great relevance to the development of new materials for photocatalysis, photon upconversion, and optoelectronics where charge separation and the formation of triplet excited states play essential roles.

## Supporting Information Available

TEM images of NPLs (Figures S1 and S13); cyclic voltammograms of PID/PMIDE (Figure S2); optical spectra of reference NPLs (Figure S3); extinction coefficients of PDI/PMIDE (Figure S4); PL lifetimes of NPLs (Figure S5); photoluminescence excitation spectra (Figure S6); supplementary TA data (Figures S7–S11 and S20); fitting of exciton binding energies (Figure S12); plot of Marcus theory (Figure S14); data analysis model (Figure S15); target analysis of TA spectra (Figures S16–S19); fitting parameters of global and target analysis (Tables S1 and S2); syntheses of perylene derivatives, estimation of the exciton binding energy and difference in CT driving force; estimation of the average number of molecules on each NPLs; single-wavelength fitting for NPLs growth excited at 490 nm; global and target analysis (PDF)
