# Supplementary material for: Tuning the Driving Force for Charge Transfer in Perovskite–Chromophore Systems
Source: J Phys Chem C Nanomater Interfaces. 2023 Jul 26;127(31):15406–15. doi: 10.1021/acs.jpcc.3c03815 (PMC10424230; doi:10.1021/acs.jpcc.3c03815)
Supplement: Supplementary file 1 — jp3c03815_si_001.pdf [file jp3c03815_si_001.pdf]

Supporting Information for

Tuning the Driving Force for Charge Transfer in Perovskite–Chromophore Systems

Zimu Wei[†]*, Jence T. Mulder*[†]*, Rajeev K. Dubey*[†,‡]*, Wiel H. Evers*[†]*, Wolter F. Jager*[†]*, Arjan J. Houtepen*[†]*, Ferdinand C. Grozema*[†,*]

[†]Department of Chemical Engineering, Delft University of Technology, Van der Maasweg 9, 2629 HZ Delft, The Netherlands.

[‡]Present address: Institut für Organische Chemie, Universität Würzburg, 97074 Würzburg, Germany.

Corresponding Author

*Ferdinand C. Grozema

E-mail: f.c.grozema@tudelft.nl

Syntheses of perylene derivatives.

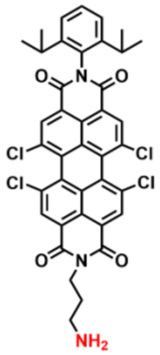

1. Synthesis of *N*-(2,6-diisopropylphenyl)-*N′*-(3-aminopropyl)-1,6,7,12-tetrachloroperylene diimide (PDI):

This compound was synthesized by following the literature procedure.[1]

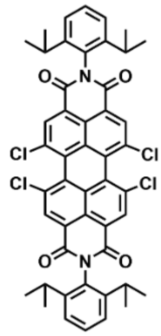

2. Synthesis of N,N′-bis(2,6-diisopropylphenyl)-1,6,7,12-tetrachloroperylene diimide (PDI-D):

This compound was synthesized by following the previously reported procedure.[2]

3. Synthesis of N-(Boc-3-aminopropyl)-1,6,7,12-tetrachloroperylene-3,4,9,10-tetracarboxy monoimide dibutylester (2):

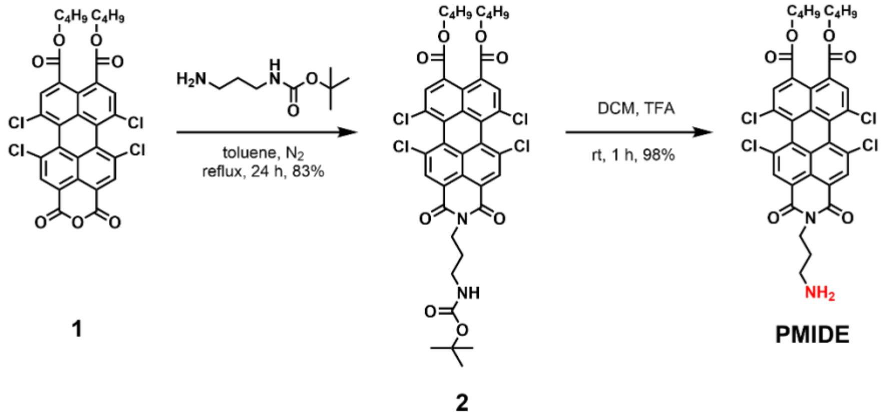

A mixture of 1,6,7,12-tetrachloroperylene monoanhydride dibutylester[3] **1** (0.50 g, 0.76 mmol, 1 eq.) and N-Boc-1,3-propanediamine (0.40 g, 2.27 mmol, 3 eq.) was taken in a round-bottom flask (50 ml) equipped with a water condenser. To this mixture, toluene (14 ml) was added. The combined mixture was refluxed for 24 h under argon atmosphere and then cooled to room temperature. Toluene was evaporated under vacuum and the solid residue was washed with water and methanol. Subsequently, the solid residue was dried and chromatographed on silica, with CH_2Cl_2 to afford the desired product (0.51 g, 83%) as an orange powder. ^1H NMR (400 MHz, $CDCl_3$): δ = 8.62 (s, 2H), 8.12 (s, 2H), 5.14 (br s, 1H), 4.41–4.32 (m, 4H), 4.28 (t, J =

8.0 Hz, 2H), 3.23–3.11 (m, 2H), 1.97–1.89 (m, 2H), 1.80 (q, J = 8.0 Hz, 4H), 1.53–1.46 (m, 4H), 1.44 (s, 9H), 1.00 ppm (t, J = 8.0 Hz, 6H). ^{1}H NMR spectrum of compound **2** in $CDCl_3$ is shown below.

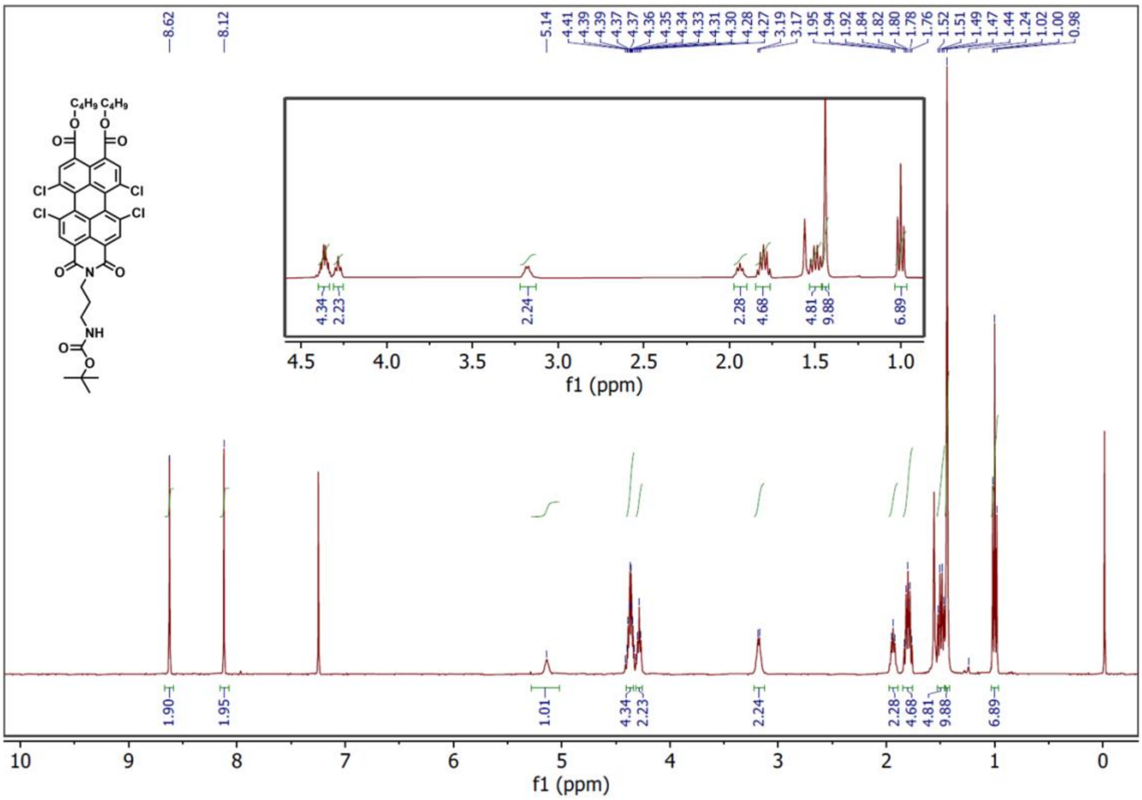

4. Synthesis of N-(3-aminopropyl)-1,6,7,12-tetrachloroperylene-3,4,9,10-tetracarboxy monoimide dibutylester (PMIDE):

Compound **2** (0.50 g, 0.61 mmol) was dissolved in DCM (10 mL) in a round-bottom flask (50 ml). Trifluoroacetic acid (3 mL) was added to this solution. The combined reaction mixture was stirred for 1 h at room temperature. The progress of the reaction was thoroughly followed by TLC analysis of removed aliquots (50:1 DCM-EtOH). After complete consumption of the starting material, more DCM (50 mL) was added. The resultant solution was washed first with aqueous K_2CO_3 and then with water. The organic phase was collected and concentrated. The crude product was then chromatographed on silica with 20:1 DCM-EtOH mixture to yield the pure product (0.43 g, 98%) as an orange powder. ^{1}H NMR (400 MHz, $CDCl_3$): δ = 8.62 (s, 2H), 8.12 (s, 2H), 4.43–4.25 (m, 6H), 2.80(br s, 2H), 1.95–1.86 (m, 2H), 1.85–1.74 (q, J = 8.0 Hz, 4H), 1.53–1.43 (m, 6H), 1.00 ppm (t, J = 8.0 Hz, 6H). ^{1}H NMR spectrum of compound **PMIDE** in $CDCl_3$ is shown below (Note: the multiplet at ca. 1.5 ppm overlaps with the water peak).

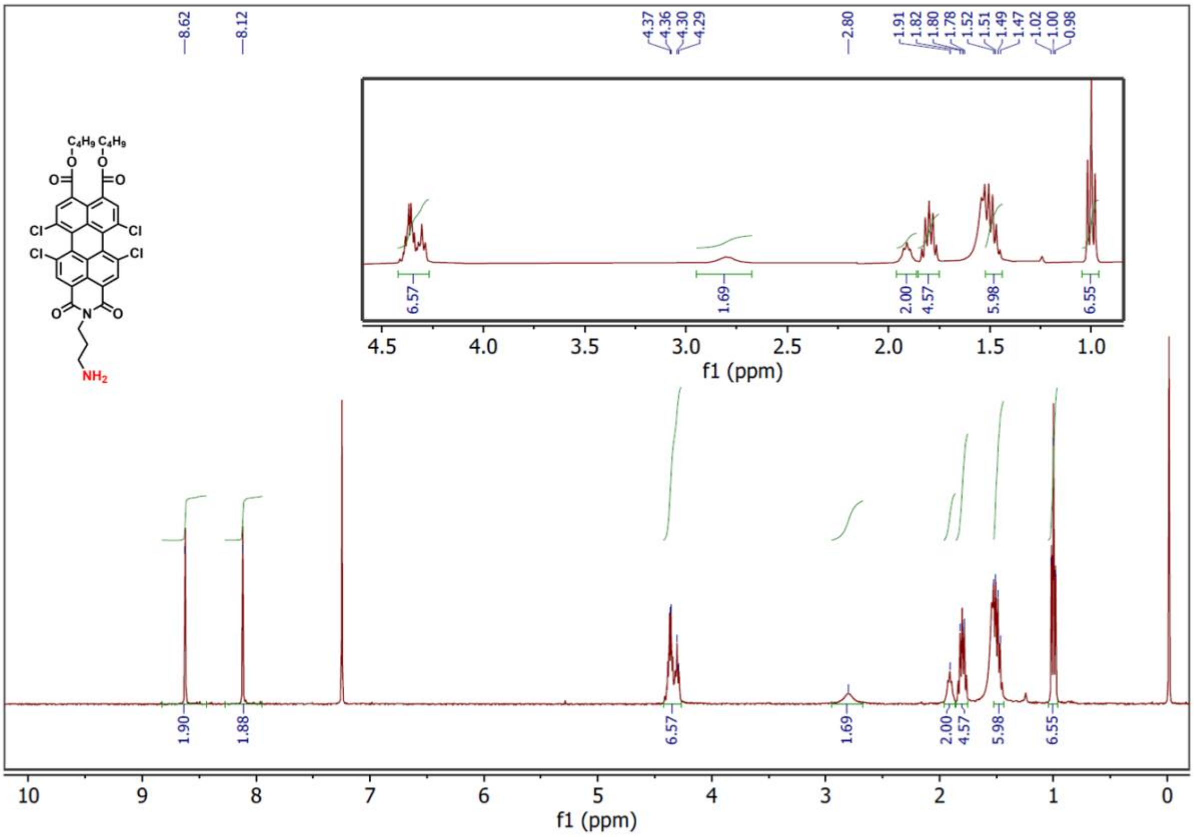

Figures and tables

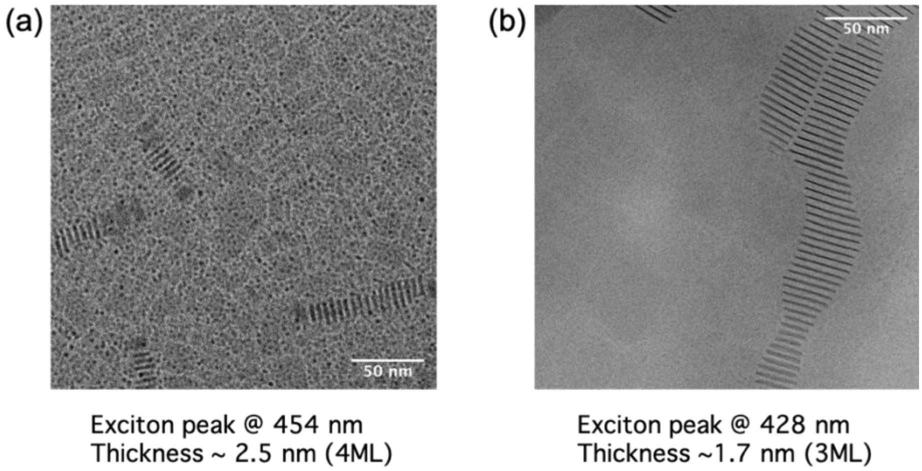

Figure S1. TEM/HR-TEM images of NPLs thickness. (a) TEM image of NPLs with exciton peak absorption at 454 nm. (b) HR-TEM image of NPLs with exciton peak absorption at 428

nm. Note that the black dots in (a) are likely to be reduced lead due to beam sensitivity of the sample.

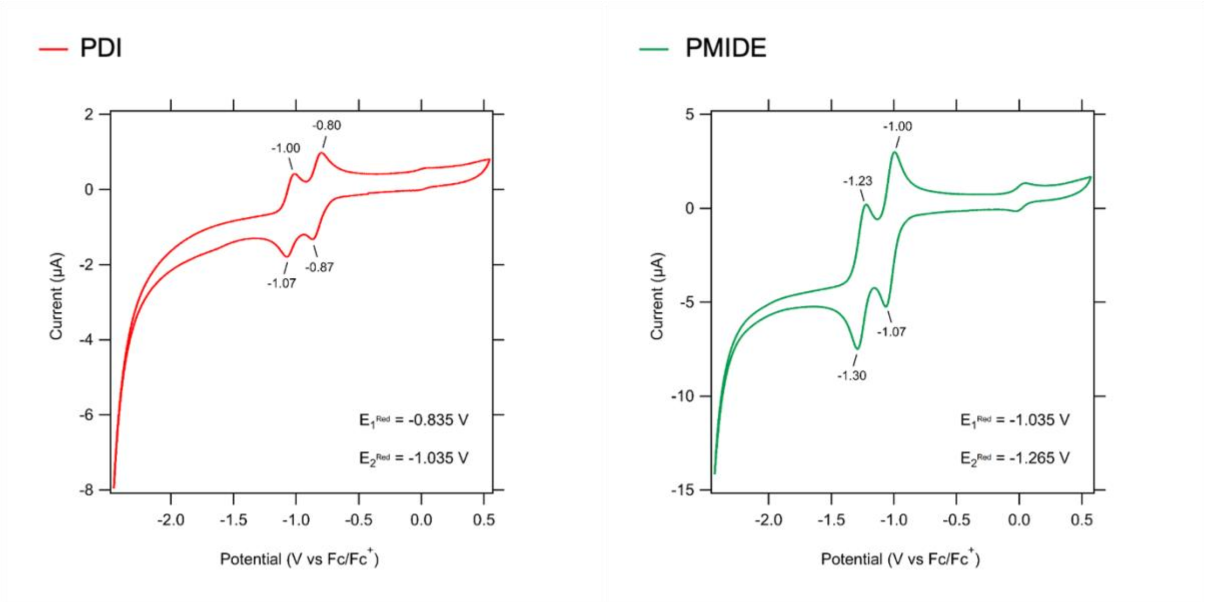

Figure S2. Cyclic voltammograms (vs Fc/Fc$^+$) of PDI and PMIDE in DCM with 0.1M $TBAPF_6$. Note that a small amount of ferrocene is added to these measurements (the small signal at 0V) as internal standard/calibration

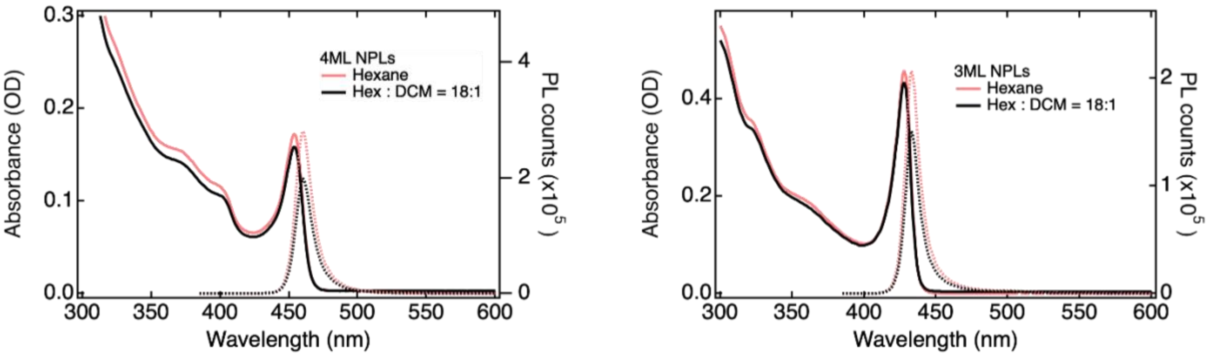

Figure S3. Optical absorption and photoluminescence emission spectra of reference NPLs before and after the addition of 150 μL DCM. PL spectra were measured with excitation at 380 nm.

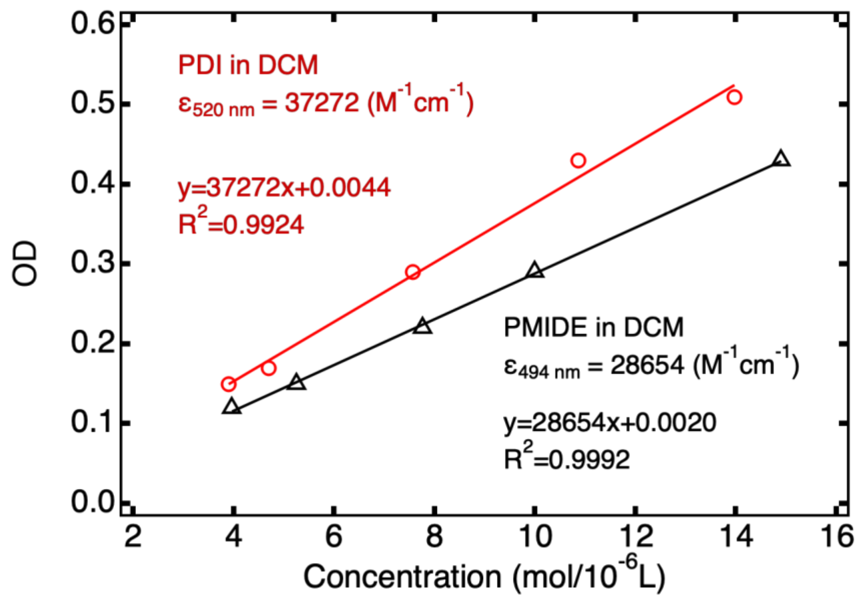

Figure S4. Extinction coefficients of PDI and PMIDE measured in DCM.

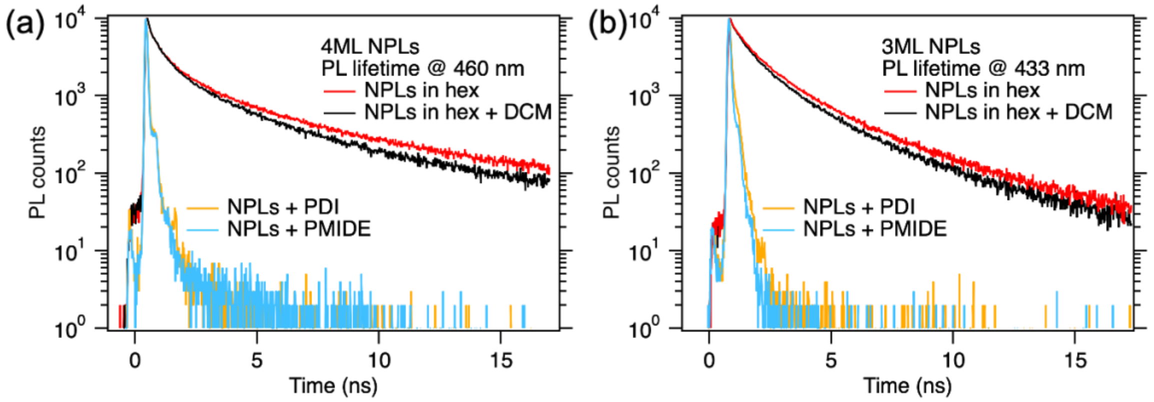

Figure S5. PL lifetime of (a) 4ML NPLs and (b) 3ML NPLs with excitation at 404 nm.

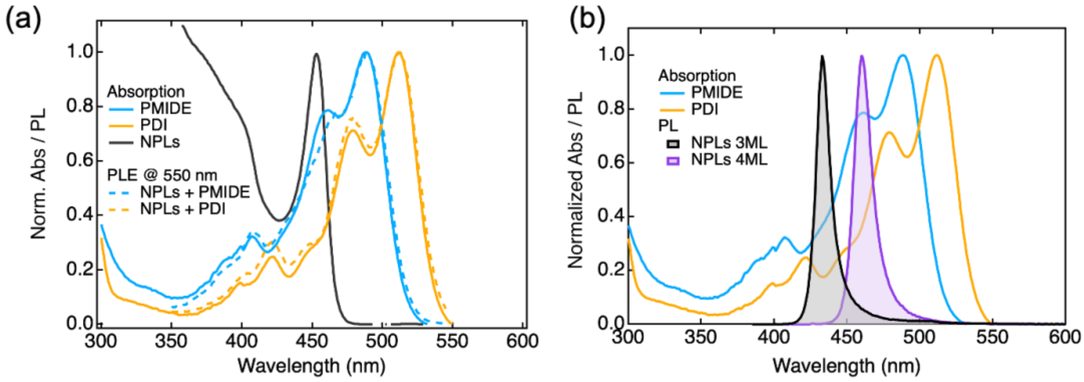

Figure S6. (a) Normalized absorption spectra of 4ML NPLs, PDI and PMIDE in Hex/DCM mixture and photoluminescence excitation spectra of 4ML NPLs + PDI/PMIDE hybrids

monitored at 550 nm. (b) Normalized photoluminescence emission spectra of NPLs and absorption spectra of PDI/PMIDE in Hex/DCM mixture.

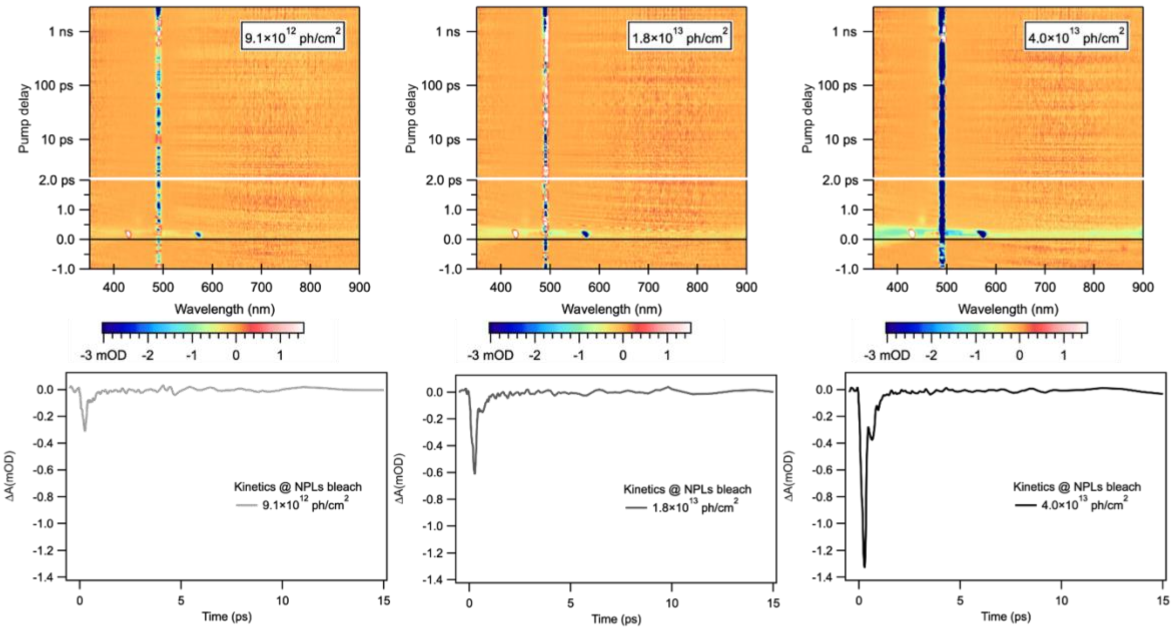

Figure S7. TA spectra of reference 4ML NPLs Hex:DCM (18:1) mixture excited at 490 nm. Upper panel: 2D TA spectra recorded at different photon fluences: 9.1×10^{12} photons/cm^2/pulse, 1.8×10^{13} photons/cm^2/pulse and 4.0×10^{13} photons/cm^2/pulse. Lower panel: TA kinetics due to coherent artifacts at exciton bleach for corresponding photon fluences.

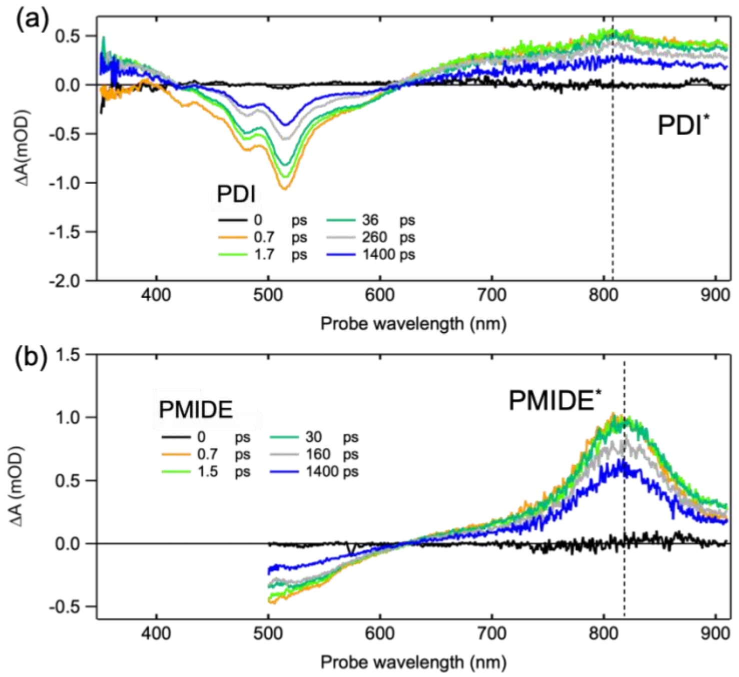

Figure S8. TA spectra of reference acceptor molecules in Hex/DCM mixture. (a) TA spectra of reference PDI excited at 510 nm. (b) TA spectra of reference PMIDE excited at 490 nm. Note that the probe light for measuring PMIDE was generated using a sapphire crystal.

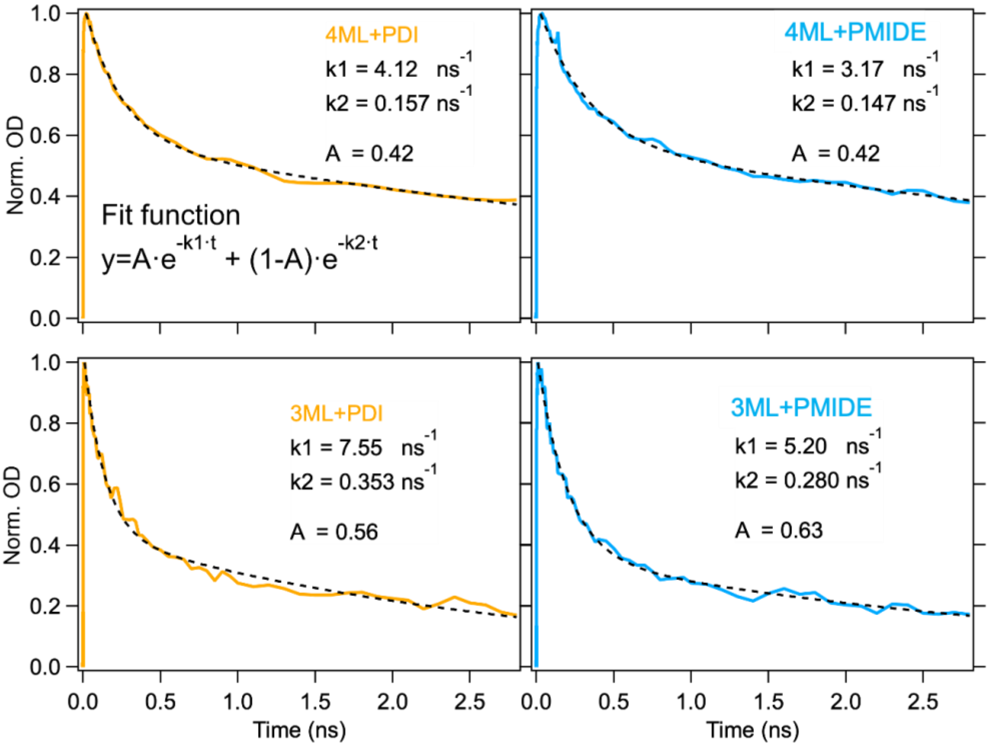

Figure S9. Normalized decay of hole bleach of NPLs and their single-wavelength fits.

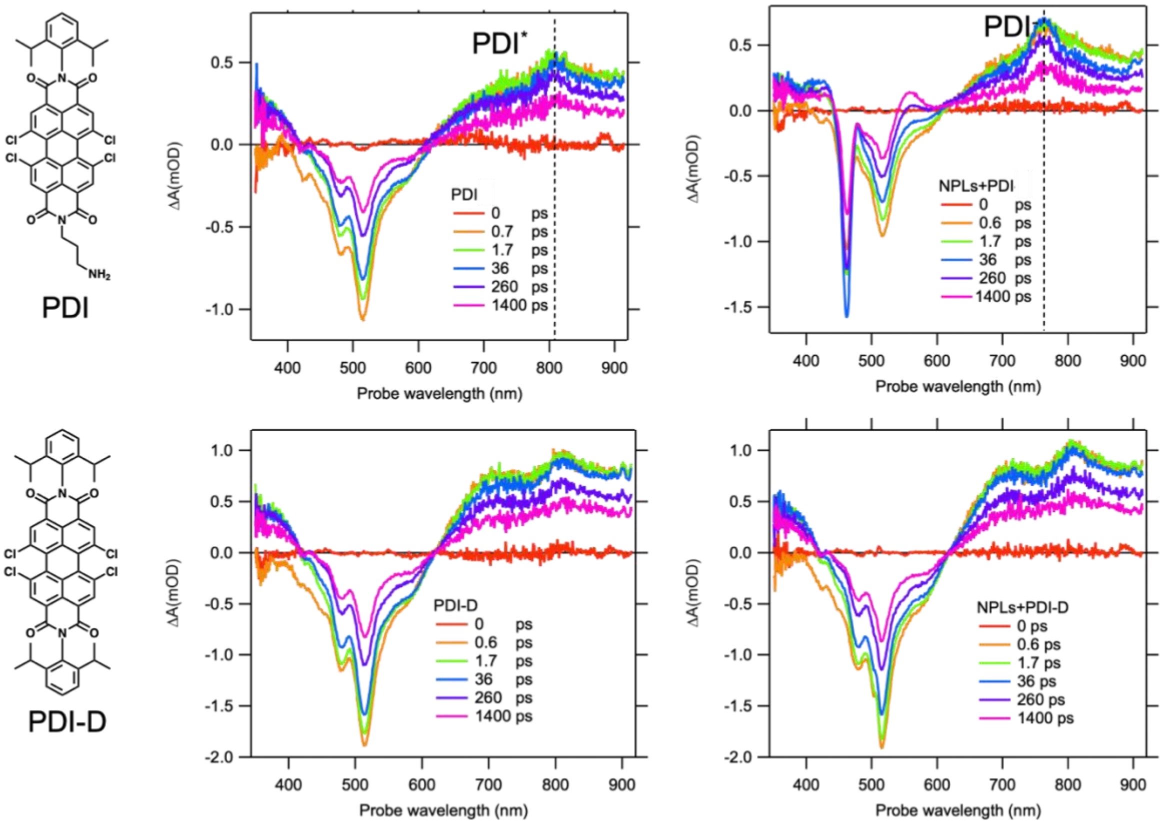

Figure S10. Transient absorption spectra excited at 510 nm. Upper panel: molecular structure of PDI (PDI used in the main text), TA spectra of PDI and of NPLs + PDI. Lower panel: molecular structure of reference PDI-D, TA spectra of PDI-D and of NPLs + PDI-D.

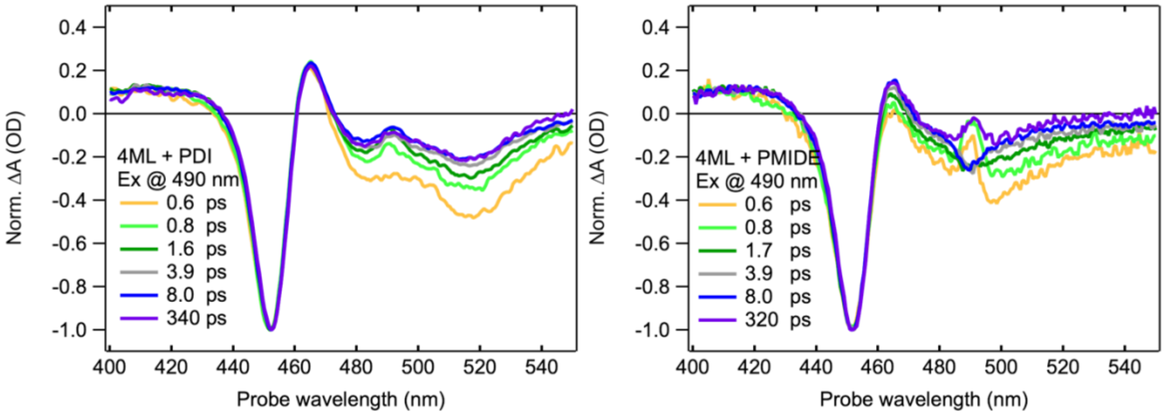

Figure S11. TA spectra normalized to the bleach of 4ML NPLs upon excitation at 490 nm in hybrid systems.

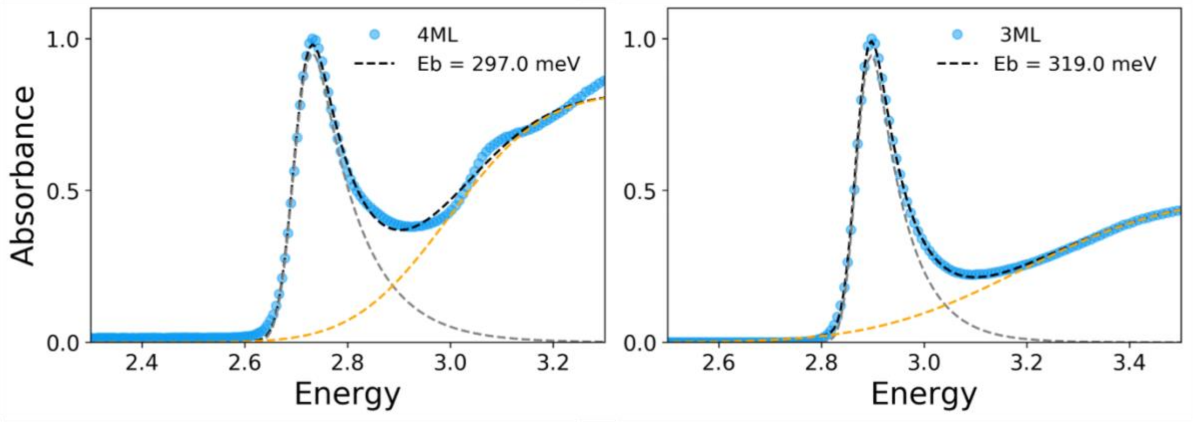

Figure S12. Normalized absorption spectra of 4ML and 3ML NPLs (blue spheres) and their fits. The black line is the fitted model, and the gray and orange lines are the exciton and continuum contributions, respectively.

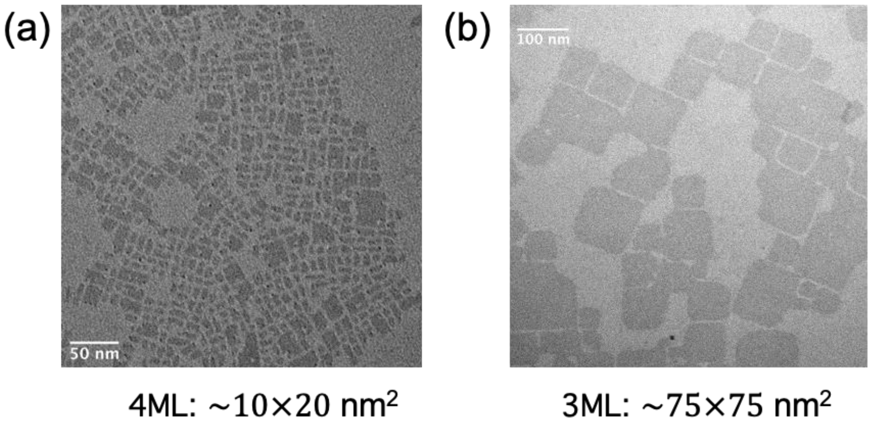

Figure S13. TEM images of NPLs lateral size drop-casted on the day of TA measurement. (a) TEM image of 4ML NPLs. (b) TEM image of 3ML NPLs

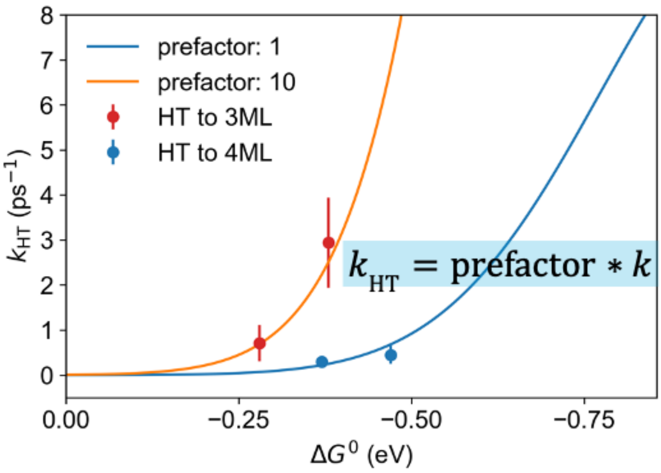

Figure S14. An example of driving force dependence of hole transfer rate for different prefactors. Red and blue dots represent hole transfer rates obtained by kinetic fitting. The relatively difference in the HT driving force was calculated based on the VB shift between 3ML and 4ML and the difference in HOMO between PDI and PMIDE. Due to uncertainty of VB energy levels for NPLs, the absolute value of driving force is set arbitrary with an upper limit of 0.8 eV. The values used: λ=1 eV, H_{DA}=200 cm^{-1}.

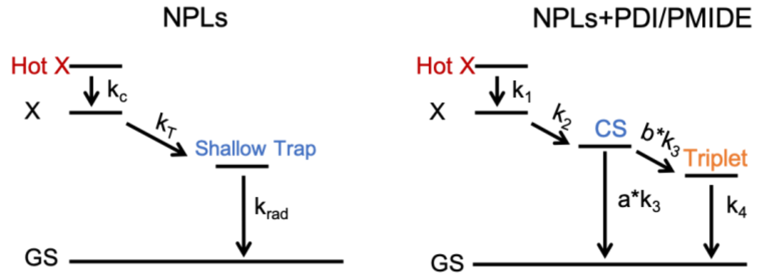

Figure S15. Global and target analysis models.

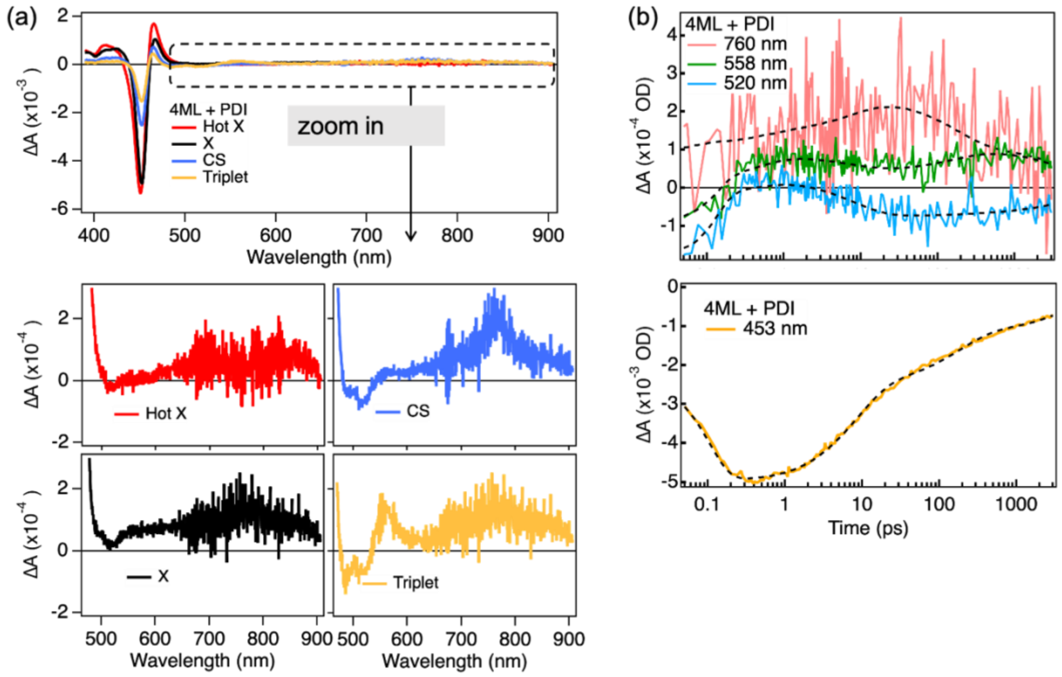

Figure S16. Target analysis of transient absorption spectra excited at 380 nm. (a) An example of species-associated spectra for 4ML + PDI. (b) Temporal kinetics and fits of 4ML + PDI at different wavelengths.

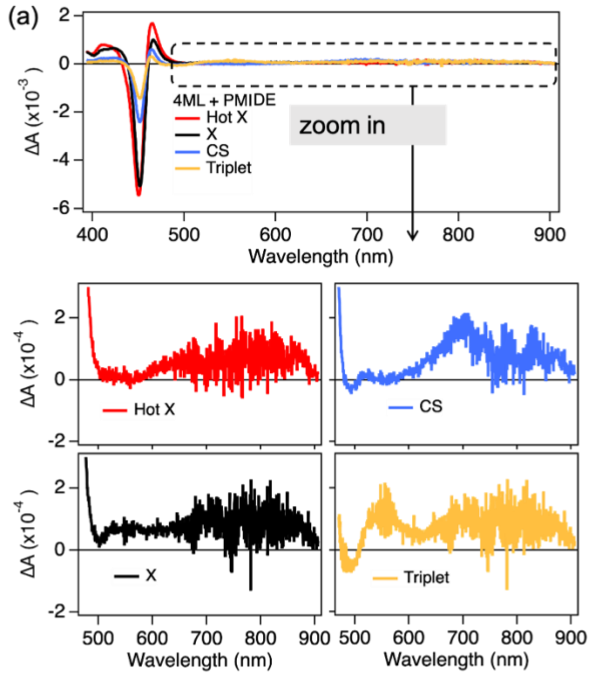

Figure S17. Target analysis of transient absorption spectra excited at 380 nm. (a) An example of species-associated spectra for 4ML + PMIDE. (b) Temporal kinetics and fits of 4ML + PMIDE at different wavelengths.

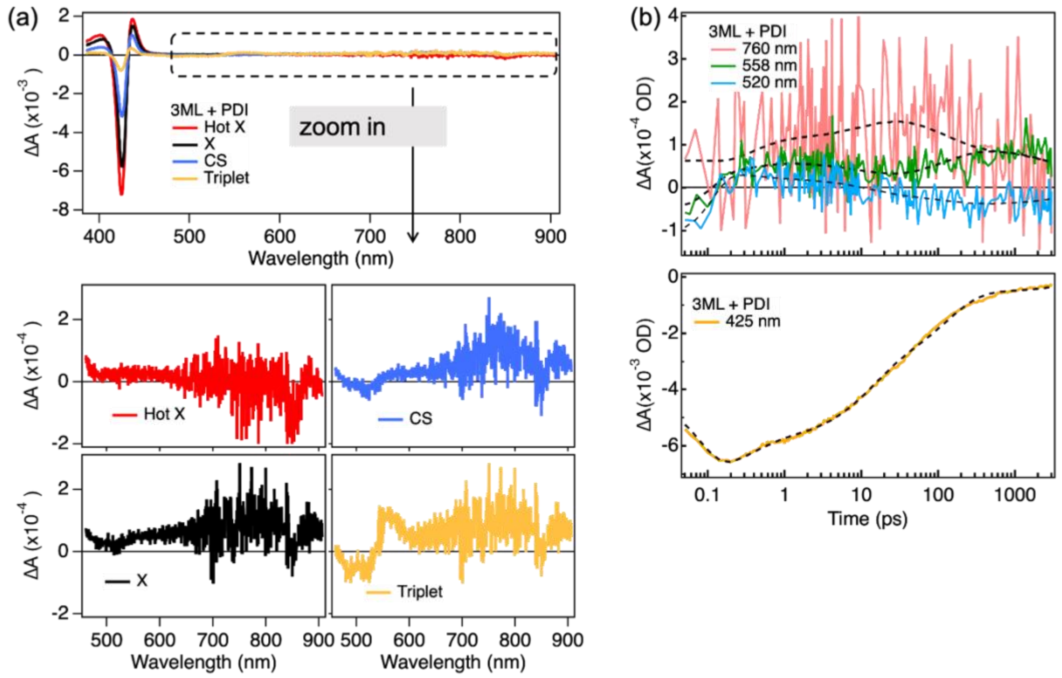

Figure S18. Target analysis of transient absorption spectra excited at 380 nm. (a) An example of species-associated spectra for 3ML + PDI. (b) Temporal kinetics and fits of 3ML + PDI at different wavelengths.

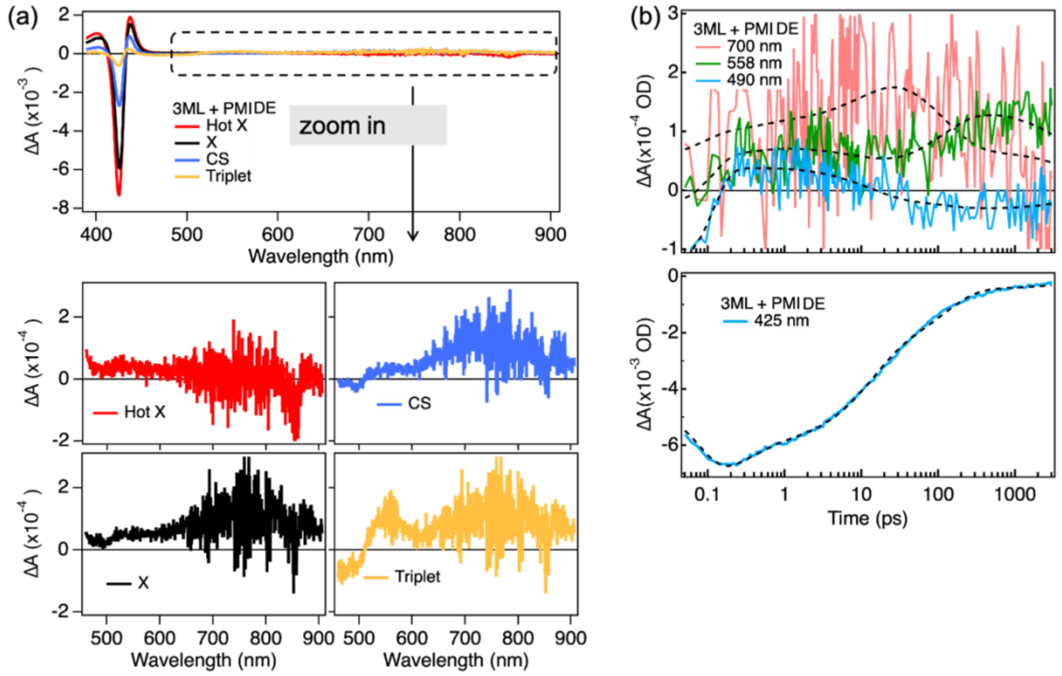

Figure S19. Target analysis of transient absorption spectra excited at 380 nm. (a) An example of species-associated spectra for 3ML + PMIDE. (b) Temporal kinetics and fits of 3ML + PMIDE at different wavelengths.

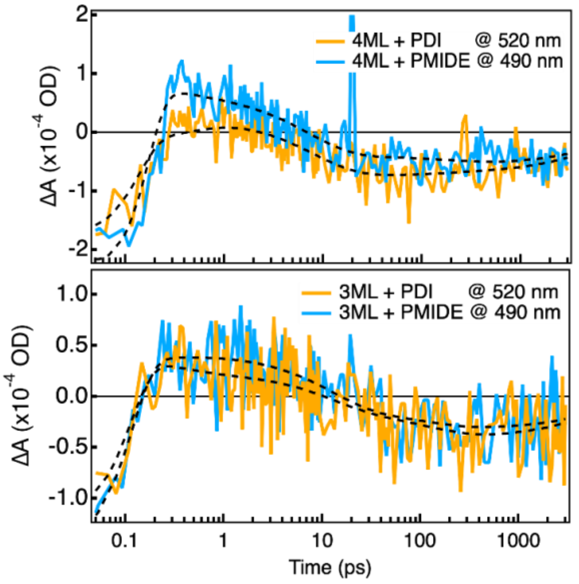

Figure S20. Comparison of the GSB kinetics for PDI and PMIDE in hybrid systems.

Table S1. Fitting parameters of global analysis

	4ML NPLs	3ML NPLs
k_c ($\tau_c$in ps)	1.61 (0.62)	1.57 (0.64)
k_T (τ_T in ps)	0.071 (14.0)	0.034 (29.4)
k_{rad} (τ_{rad} in ns)	0.00040 (2.48)	0.00062 (1.62)

Table S2. Fitting parameters of target analysis

	4ML+PDI	4ML+PMIDE	3ML+PDI	3ML+PMIDE
k_1 ($\tau_1$in ps)	2.12 (0.47)	2.08 (0.48)	2.81 (0.35)	2.73 (0.36)
k_2 (τ_2 in ps)	0.124 (8.08)	0.147 (6.78)	0.0847 (11.73)	0.0846 (11.83)
k_3 (τ_3 in ps)	0.0066 (151)	0.0064 (156)	0.0085 (118)	0.0094 (107)
k_4 ($\tau_4$in ps)	0.00019 (5196)	0.00021 (4701)	0.00021 (4825)	0.00016 (6250)
a	0.2	0.2	0.25	0.25
b	0.8	0.8	0.75	0.75

Estimation of the exciton binding energy and difference in CT driving force

To have an approximate estimation of the exciton binding energy of NPLs, their absorption spectra in hexane were normalized and fitted with a quantum-well absorption model[4,5], which has been widely used for extracting exciton binding energy in $CsPbBr_3$ NPLs[6–8]. According to this model, the absorption spectrum $\mathrm{A}(E)$ is described as a sum of the exciton peak absorption, $\mathrm{X}(E)$ and continuum band absorption $\mathrm{Con}(E)$:

$$\mathrm{A}(E) = \mathrm{X}(E) + \mathrm{Con}(E),$$

where

$$\mathrm{X}(E) = \frac{1}{2\eta}\left[\mathrm{erf}\left\{\frac{E-E_0}{\gamma_x} - \frac{\gamma_x}{2\eta}\right\} + 1\right]\exp\left(\frac{\gamma^2}{4\eta^2} - \frac{E-E_0}{\eta}\right)$$

and

$$\mathrm{Con}(E) = \frac{H_c}{2}\left[\mathrm{erf}\left\{\frac{E-E_0-E_b}{\gamma_c}\right\} + 1\right].$$

E_0, E_b, γ_x, γ_c, η, and H_c represent absolute exciton energy, exciton binding energy, exciton peak width, continuum edge width, asymmetric broadening of exciton peak and the step height of continuum edge, respectively.

As shown in **Figure S12**, the fitting results in the exciton binding energies of 300 ± 30 meV for 4ML NPLs and 320 ± 20 meV for 3ML NPLs, which a in agreement with reported values ranging from 260 to 350 meV[7,8].

With estimated exciton binding energies, E_b, and exciton energy, E_x, obtained from absorption spectra, the energy shift in VB and CB from 4ML to 3ML NPLs was calculated according to:[9]

$$\Delta E_{\mathrm{VB}} = \frac{m_e}{m_e + m_h}\left[\left(E_{x,3\mathrm{ML}} + E_{b,3\mathrm{ML}}\right) - \left(E_{x,4\mathrm{ML}} + E_{b,4\mathrm{ML}}\right)\right]$$

and

$$\Delta E_{\mathrm{CB}} = \frac{m_h}{m_e + m_h}\left[\left(E_{x,3\mathrm{ML}} + E_{b,3\mathrm{ML}}\right) - \left(E_{x,4\mathrm{ML}} + E_{b,4\mathrm{ML}}\right)\right],$$

where m_e and m_h represent the effective mass for electron and hole of $CsPbBr_3$ NPLs. Since similar effective mass has been reported for electron and hole of $CsPbBr_3$ NPLs regardless of their thickness.[10] The CB and VB shift from 4ML to 3ML is estimated to be the same at 95 meV.

Estimation of the average number of molecules on each NPLs

It has been reported that at high photon energies the intrinsic absorption coefficient of CsPbBr3 nanocrystals is size-independent.[11] This means based on the absorbance of NPLs at high photon energies, such as at 335 nm, the total volume of NPLs in solution can be estimated. With average lateral size and thickness of NPLs obtained from TEM measurements, the total number of 4ML NPLs are estimated to be 15 times larger than that of 3ML NPLs. Since both NPLs lead to similar PLQY for each acceptor molecule (**Figure 2c** and **2f**), it is reasonable to assume that the total amount of molecules attached are similar for both NPLs. Accordingly, the average number of molecules attached on 3ML NPLs should be 15 times larger than that of 4ML NPLs.

Kinetics fitting

Single-wavelength fitting for NPLs growth excited at 490 nm.

As the formation rate of the NPLs bleach directly represents the hole transfer rate, the dynamics at the wavelengths of the main exciton bleach for NPLs were fitted to extract the hole transfer rate. Specifically, the obtained dynamics are considered as a convolution of the real dynamics of the sample by the Instrument Response Function (IRF) of the setup. Hence, to extract the rates from the real dynamics, the TA data was fitted by an analytical form for the convolution of a function describing hole transfer by a function describing the IRF[12].

The IRF function was described by a Gaussian function:

$$IRF\ (t, t_0, \sigma) = e^{-\frac{(t-t_0)^2}{2\sigma^2}}.$$

The NPL growth was described by a step-function multiplied by an exponential ingrowth of a fraction of the signal:

$$NPL(t, t_0, \tau, f_a) = \theta(t, t_0)\left(1 - f_a \cdot e^{-\frac{t-t_0}{\tau}}\right),$$

where θ is the Heaviside function and $(1 - f_a)$ accounts for the initial signal at t_0 due to coherent artifact (**Figure S7**).

The convolution of these two function yields:

$$F(t, t_0, \sigma, \tau, f_a, A) = \frac{A}{2}\left(1 + \text{erf}\left(\frac{t-t_0}{\sqrt{2}\sigma}\right) - f_a \cdot \exp\left(\frac{\sigma^2}{2\tau^2} - \frac{t-t_0}{\tau}\right) \cdot \left(1 + \text{erf}\left(\frac{t-t_0}{\sqrt{2}\sigma} - \frac{\sigma}{\sqrt{2}\tau}\right)\right)\right),$$

where A is a fitting parameter for normalizing the bleach amplitude.

Global and Target analysis.

The two-dimensional TA data were analyzed by global and target analysis using Glotaran[13]. With this method, the 2D data matrix, $\Psi(\lambda, t)$, is modeled as a linear combination of n components given by the equation:

$$\Psi(\lambda, t) = \sum_{l=1}^{n_{comp}} c_l(t)\varepsilon_l(\lambda)$$

Each component has its own spectrum, $\varepsilon_l(\lambda)$, that following a certain concentration profile, $c_l(t)$. To be specific, with global analysis a sequential kinetic model is used to describe the evolution of one component into the other with increasing time constants. In addition to the rate constants, each component is characterized by its own evolution associated difference spectrum (EADS). Although the EADS may reflect mixtures of excited species due to the simplicity of the sequential model, the global analysis does provide important information on the temporal evolution of the system. For more complex systems, that following non-sequential kinetics, the target analysis with a specific kinetic model were be used. The resulting species associated difference spectra (SADS) should represent the true spectra of the individual excited species based on the used kinetic model.

Global analysis of excited state kinetics of $CsPbBr_3$ NPLs excited at 380 nm

In order to adequately fit the excited state kinetics of CsPbBr3 NPLs, a three-step sequential model was used (**Figure S14**). Based on this model, upon photoexcitation above the band gap of NPLs, the hot exciton relaxes to form the band-edge exciton with a cooling rate constant, k_c. Subsequently, the short lifetime on the order of tens of picosecond is assigned to the shallow trapping, k_T, followed by a longer lifetime of a few nanoseconds as the radiative recombination lifetime, k_{rad}. Fitting parameters are listed in **Table S1**.

Target analysis of excited state kinetics of NPLs+PDI/PMIDE systems excited at 380 nm

To model the excited state kinetics of the hybrid systems, we assume each NPL is attached with a PDI/PMIDE as the concentration of acceptor molecules is much higher than the concentration of NPLs. In order to keep the kinetic model as simple as possible, a four-step

sequential model was firstly used: Hot X → X → CS state → triplet state → GS. However, with this sequential model, the characteristics of PDI/PMIDE anion absorption was found in the decay-associated spectra of the triplet state. In order to obtain a better species-associated spectra of the triplet state, a brunched model (target analysis) was used (**Figure S14**). In this model, a small fraction of CS state is allowed to decay directly to the ground state without forming the triplet states. The spectra of second and forth species in the NIR region are forced to be the same, in order to reduce the contamination of anion absorption in the triplet spectra. Despite the difference in the global and target analysis models, the first (cooling of hot exciton) and second (formation of CS state) rate constants are very similar in both sequential and brunched models. Note that the second rate constant (k_2) will not only be influenced by the charge transfer rate but also, to some extent, the fast trapping in the NPLs. In case of energy transfer followed by the ultrafast hole transfer, k_2 should reflect the total contribution of both processes. Fitting parameters are listed in **Table S2**.

Reference

(1) Gélvez-Rueda, M. C.; Fridriksson, M. B.; Dubey, R. K.; Jager, W. F.; van der Stam, W.; Grozema, F. C. Overcoming the Exciton Binding Energy in Two-Dimensional Perovskite Nanoplatelets by Attachment of Conjugated Organic Chromophores. *Nat. Commun.* **2020**, *11*, 1901.

(2) Dubey, R. K.; Westerveld, N.; Grozema, F. C.; Sudhölter, E. J. R.; Jager, W. F. Facile Synthesis of Pure 1,6,7,12-Tetrachloroperylene-3,4,9,10-Tetracarboxy Bisanhydride and Bisimide. *Org. Lett.* **2015***, 17,* 1882–1885.

(3) Dubey, R. K.; Westerveld, N.; Sudhölter, E. J. R.; Grozema, F. C.; Jager, W. F. Novel Derivatives of 1,6,7,12-Tetrachloroperylene-3,4,9,10-Tetracarboxylic Acid: Synthesis, Electrochemical and Optical Properties. *Org. Chem. Front.* **2016**, *3*, 1481–1492.

(4) Grim, J. Q.; Christodoulou, S.; Di Stasio, F.; Krahne, R.; Cingolani, R.; Manna, L.; Moreels, I. Continuous-Wave Biexciton Lasing at Room Temperature Using Solution-Processed Quantum Wells. *Nat Nanotechnol* **2014**, *9*, 891-895.

(5) Naeem, A.; Masia, F.; Christodoulou, S.; Moreels, I.; Borri, P.; Langbein, W. Giant Exciton Oscillator Strength and Radiatively Limited Dephasing in Two-Dimensional Platelets. *Phys. Rev. B Condens. Matter Mater. Phys.* **2015**, *91*, 121302.

(6) Peng, S.; Wei, Q.; Wang, B.; Zhang, Z.; Yang, H.; Pang, G.; Wang, K.; Xing, G.; Sun, X. W.; Tang, Z. Suppressing Strong Exciton–Phonon Coupling in Blue Perovskite Nanoplatelet Solids by Binary Systems. *Angew. Chem. Int. Ed.* **2020**, *59*, 22156-22162.

(7) Vale, B. R. C.; Socie, E.; Burgos-Caminal, A.; Bettini, J.; Schiavon, M. A.; Moser, J. E. Exciton, Biexciton, and Hot Exciton Dynamics in CsPbBr3 Colloidal Nanoplatelets. *J. Phys. Chem. Lett.* **2020**, *11*, 387–394.

(8) Li, Q.; Lian, T. Ultrafast Charge Separation in Two-Dimensional CsPbBr 3 Perovskite Nanoplatelets. *J. Phys. Chem. Lett.* **2019**, *10*, 566-573.

(9) Wang, J.; Ding, T.; Gao, K.; Wang, L.; Zhou, P.; Wu, K. Marcus Inverted Region of Charge Transfer from Low-Dimensional Semiconductor Materials. *Nat. Commun.* **2021**, *12*, 6333.
(10) Akkerman, Q. A.; Motti, S. G.; Srimath Kandada, A. R.; Mosconi, E.; D'Innocenzo, V.; Bertoni, G.; Marras, S.; Kamino, B. A.; Miranda, L.; De Angelis, F.; et al. Solution Synthesis Approach to Colloidal Cesium Lead Halide Perovskite Nanoplatelets with Monolayer-Level Thickness Control. *J. Am. Chem. Soc.* **2016**, *138*, 1010–1016.
(11) Maes, J.; Balcaen, L.; Drijvers, E.; Zhao, Q.; De Roo, J.; Vantomme, A.; Vanhaecke, F.; Geiregat, P.; Hens, Z. Light Absorption Coefficient of CsPbBr3 Perovskite Nanocrystals. *J. Phys. Chem. Lett.* **2018**, *9,* 3093–3097.
(12) Grimaldi, G.; Geuchies, J. J.; Van Der Stam, W.; Du Fossé, I.; Brynjarsson, B.; Kirkwood, N.; Kinge, S.; Siebbeles, L. D. A.; Houtepen, A. J. Spectroscopic Evidence for the Contribution of Holes to the Bleach of Cd-Chalcogenide Quantum Dots. *Nano Lett.* **2019**, *19*, 3002-3010.
(13) Snellenburg, J. J.; Laptenok, S.; Seger, R.; Mullen, K. M.; van Stokkum, I. H. M. Glotaran: A Java-Based Graphical User Interface for the R Package TIMP. *J. Stat. Softw.* **2012**, *49*, 1-22.
